# Intercompatibility of eukaryotic and Asgard archaea ribosome-translocon machineries

**DOI:** 10.1016/j.jbc.2024.107673

**Published:** 2024-08-14

**Authors:** Isaac Carilo, Yosuke Senju, Takeshi Yokoyama, Robert C. Robinson

**Affiliations:** 1 Research Institute for Interdisciplinary Science (RIIS), Okayama University, Okayama, Japan; 2 Graduate School of Life Sciences, Tohoku University, Sendai, Japan; 3 School of Biomolecular Science and Engineering (BSE), Vidyasirimedhi Institute of Science and Technology (VISTEC), Rayong, Thailand

**Keywords:** evolution, Asgard archaea, endoplasmic reticulum, Sec61, translocon, OST complex, TRAP complex, X-ray crystallography

## Abstract

In all domains of life, the ribosome-translocon complex inserts nascent transmembrane proteins into, and processes and transports signal peptide-containing proteins across, membranes. Eukaryotic translocons are anchored in the endoplasmic reticulum, while the prokaryotic complexes reside in cell membranes. Phylogenetic analyses indicate the inheritance of eukaryotic Sec61/oligosaccharyltransferase/translocon-associated protein translocon subunits from an Asgard archaea ancestor. However, the mechanism for translocon migration from a peripheral membrane to an internal cellular compartment (the proto-endoplasmic reticulum) during eukaryogenesis is unknown. Here we show compatibility between the eukaryotic ribosome-translocon complex and Asgard signal peptides and transmembrane proteins. We find that Asgard translocon proteins from *Candidatus* Prometheoarchaeum syntrophicum strain Candidatus Prometheoarchaeum syntrophicum strain MK-D1, a Lokiarchaeon confirmed to contain no internal cellular membranes, are targeted to the eukaryotic endoplasmic reticulum on ectopic expression. Furthermore, we show that the cytoplasmic domain of Candidatus Prometheoarchaeum syntrophicum strain MK-D1 oligosaccharyltransferase 1 (ribophorin I) can interact with eukaryotic ribosomes. Our data indicate that the location of existing ribosome-translocon complexes, at the protein level, determines the future placement of yet-to-be-translated translocon subunits. This principle predicts that during eukaryogenesis, under positive selection pressure, the relocation of a few translocon complexes to the proto-endoplasmic reticulum will have contributed to propagating the new translocon location, leading to their loss from the cell membrane.

Signal peptides (SPs) and transmembrane domains direct membrane targeting, integration, or translocation in all domains of life (1, 2, 3, 4, 5, 6). In co-translational translocation, the nascent polypeptide containing either an N-terminal SP or a transmembrane helix is recognized by the signal recognition particle (SRP) as it emerges from the ribosome exit tunnel (7, 8, 9, 10, 11). The SRP-ribosome-nascent chain complex is targeted to the appropriate membrane through interactions with an SRP receptor and between the ribosome and SecY/Sec61 translocon (12). This triggers the transfer of the nascent chain to the SecY/Sec61 translocon complex and SRP dissociation (13, 14, 15). SPs are proteolytically cleaved by membrane-bound signal peptidase complexes on the opposite side of the membrane to ribosome engagement (16). In eukaryotes, post-translational translocation can also occur, whereby fully synthesized proteins are inserted into the endoplasmic reticulum (ER) membrane (17).

In eukaryotes, the core Sec61 channel forms a larger translocon associating with the multimeric translocon-associated protein (TRAP) complex and oligosaccharyltransferase (OST) complexes (18). In the Sec61/OST/TRAP translocon, the TRAP complex aids ribosome docking to the Sec61 translocon and participates in protein folding (19), while the OST complex mediates N-linked glycosylation (20). The TRAP complex is not found in bacteria, and the bacterial oligosaccharyltransferase is a single polypeptide chain. Understanding these differences and the emergence of the eukaryotic Sec61/OST/TRAP translocon are key questions for the eukaryogenesis field.

There are many hypotheses for the origin of eukaryotic internal membranes (21, 22, 23, 24, 25, 26). However, it is challenging to reproduce the actual events that occurred during eukaryogenesis, which occurred more than 1.8 billion years ago (27), or to design meaningful experiments that can support a particular theory. Possibly, the best tools available for understanding the emergence of eukaryotic internal membranes are the transmembrane proteins associated with each membrane. In particular, the eukaryotic Sec61/OST/TRAP translocon resides in the ER, however, in prokaryotes, the homologous SecYEG translocon sits in the cell membrane. While both translocons are transverse membranes, they differ in their specific membrane localization. This distinction provides an opportunity to explore how the Sec61 translocon may have undergone relocalization during eukaryogenesis. Phylogenetic analyses of the preprotein translocase channel SecY/Sec61 and the OST complex catalytic subunit (STT3) indicate that the eukaryotic versions of these translocon subunits are more closely related to their Asgard archaea counterparts than to other prokaryotic homologs (28, 29). Asgard archaea are predicted to have a complete Sec61αβγ complex and many of the components of the OST and TRAP complexes (30). Here we explore the compatibility of Asgard translocon proteins from *Candidatus* Prometheoarchaeum syntrophicum strain Candidatus Prometheoarchaeum syntrophicum strain MK-D1 (MK-D1) (21) on expression in mammalian cells.

## Results

### Asgard translocon signal sequence proteins

Sequence searches against the MK-D1 genome predict genes for the entire Sec61αβγ complex; OST1 (ribophorin), OST3/6, and STT3 from the OST complex; and TRAP subunits α, β, and γ (Table S1). Similar searches against sequence databases, incorporating all domains of life, predict: the TRAP subunits are restricted to Asgard archaea and eukaryotes; OST1 and OST3/6 subunits are found in eukaryotes, and TACK and Asgard archaea; and the Sec61αβγ/Sec61YEG and STT3 subunits are universal. MK-D1 also possesses typical genes for archaeal SRP proteins (31): SRP19, SRP54, and SRP docking protein FtsY (Table S1).

In order to probe the similarities between the MK-D1 and eukaryotic preprotein signal sequences, we analyzed the entire MK-D1 genome-predicted protein sequences with the signal sequence prediction software SignalP 6.0 (32). Many MK-D1 transmembrane proteins, including OST1, TRAPα, and TRAPβ, were predicted to have eukaryotic-like signal sequences with probabilities of 0.77, 1.00, and 0.99, respectively; These results are comparable with the probabilities for the human proteins of 1.00, 0.81 and 1.00, respectively (Table 1). However, the eukaryotic subcellular localization software DeepLoc 2.0 (33) predicted the localization of these MK-D1 membrane proteins to a variety of possible eukaryotic membranes in comparison to the strong ER localization predicted for the human translocon subunits (Table 1), which is in line with the evolution of complex membrane protein processing within eukaryotic cells. SignalP 6.0 did not predict signal peptides for the homologs of eukaryotic ER chaperones (34) in MK-D1, or in any other Asgard organism, indicating that the Asgard chaperone homologs are cytoplasmic.

**Table 1:** Signal peptide and eukaryotic cell localization predicted for human and MK-D1 translocon components

Protein	Human					MK-D1				
	SP	CM	ER	L/V	Golgi	SP	CM	ER	L/V	Golgi
OST1	1.00	0.2974	**0.8539**	0.3249	0.5281	0.77	0.4928	0.4491	**0.5406**	0.3896
OST3/6	-	0.4104	**0.8222**	0.4776	0.7766	-	0.2012	**0.6849**	0.3375	0.4546
STT3	-	0.2676	**0.9103**		0.5628	-	0.3056	**0.5482**	0.2918	0.2968
TRAP-α	1.00	0.2115	**0.9329**	0.6124	0.7756	1.00	0.6247	0.4706	**0.6728**	0.3788
TRAP-β	1.00	0.1857	**0.8883**	0.2093	0.3706	1.00	**0.5493**	0.2311	0.5200	0.2683
TRAP-γ	-	0.1407	**0.9088**	0.1638	0.6022	-	0.1468	0.5100	**0.6175**	0.2737
Sec61α	-	0.4400	**0.5555**	0.3185	0.2664	-	0.2875	**0.5734**	0.3097	0.2100
Sec61β	0.16	0.4051	**0.8263**	0.2655	0.6076	-	0.2468	**0.6042**	0.4534	0.5575
Sec61γ	-	0.3844	**0.8462**	0.5236	0.5854	-	0.0690	0.3514	0.3797	**0.5174**
S-layer	-	-	-	-	-	0.69	**0.5185**	0.3739	0.3732	0.1124

CM, ER, L/V, and Golgi are the predicted probabilities to be localized to the cell membrane, endoplasmic reticulum, lysosome/vacuole or Golgi apparatus, respectively. S-layer protein predictions are also included for MK-D1 as a reference. Bold numbers indicate the highest probability for each protein.

Abbreviation: ER, endoplasmic reticulum; SP, signal peptide probability; MK-D1, Candidatus Prometheoarchaeum syntrophicum strain MK-D1; OST, oligosaccharyltransferase; SP, signal peptide; TRAP, translocon-associated protein.

**Figure 1:**
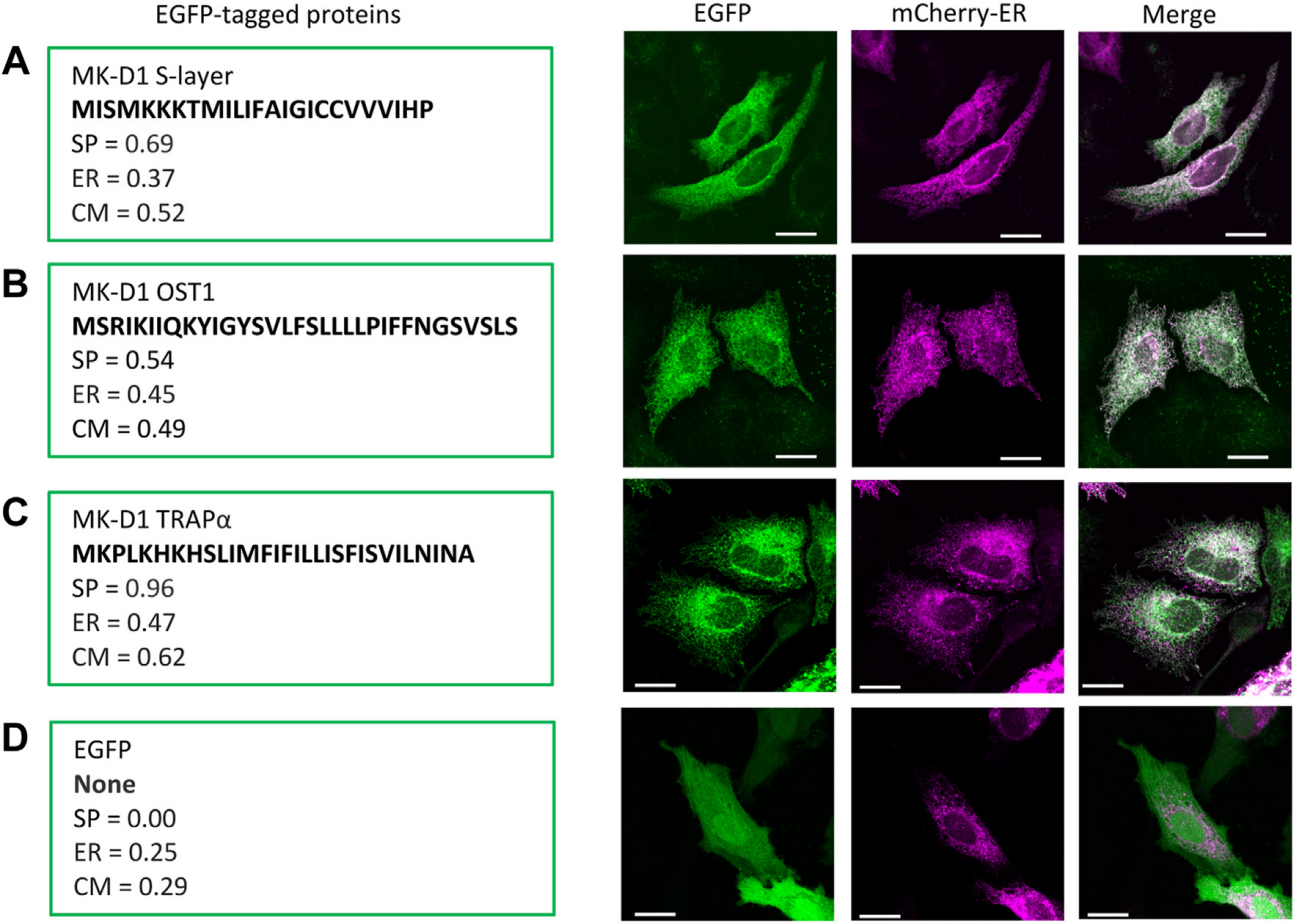
**Representative images of the localization of MK-D1 full-length signal peptide-containing proteins on transfection in HeLa cells.** Cells were co-transfected to express EGFP-fused signal peptide-containing proteins and an ER-localizing mCherry construct. At 24 h post-transfection, cells were fixed and imaged using the confocal microscope. EGFP (*green*), mCherry (*magenta*), and merged images are shown. *A*, MK-D1 S-layer protein. *B*, MK-D1 OST1. *C*, MK-D1 TRAPα. *D*, EGFP alone. The signal peptide sequences for each MK-D1 protein (*bold*), SignalP 6.0 signal peptide prediction scores (SP), and DeepLoc 2.0 localization probabilities for the endoplasmic reticulum (ER) and cell membrane (CM) are given for each EGFP construct. Scale bar = 20 μm. Quantification of the colocalization and distributions relative to the plasma membrane are found in Figs. S1 and S2. MK-D1, Candidatus Prometheoarchaeum syntrophicum strain MK-D1; OST, oligosaccharyltransferase; TRAP, translocon-associated protein.

To experimentally determine whether the MK-D1 preproteins exhibit a preferred location in eukaryotic cells, we ectopically expressed MK-D1 OST1, TRAPα, and the cell surface S-layer protein as EGFP fusion proteins in HeLa cells (Fig. 1, *A*–*C*). In all three cases, these MK-D1 cell surface proteins co-localized with a mCherry ER marker and did not localize to the cell membrane. By contrast, EGFP alone showed no co-localization with the ER marker (Fig. 1*D*). These data indicate that heterologously expressed cell surface Asgard preproteins are translated and processed at the ER, where the eukaryotic translocase is located.

**Figure 2:**
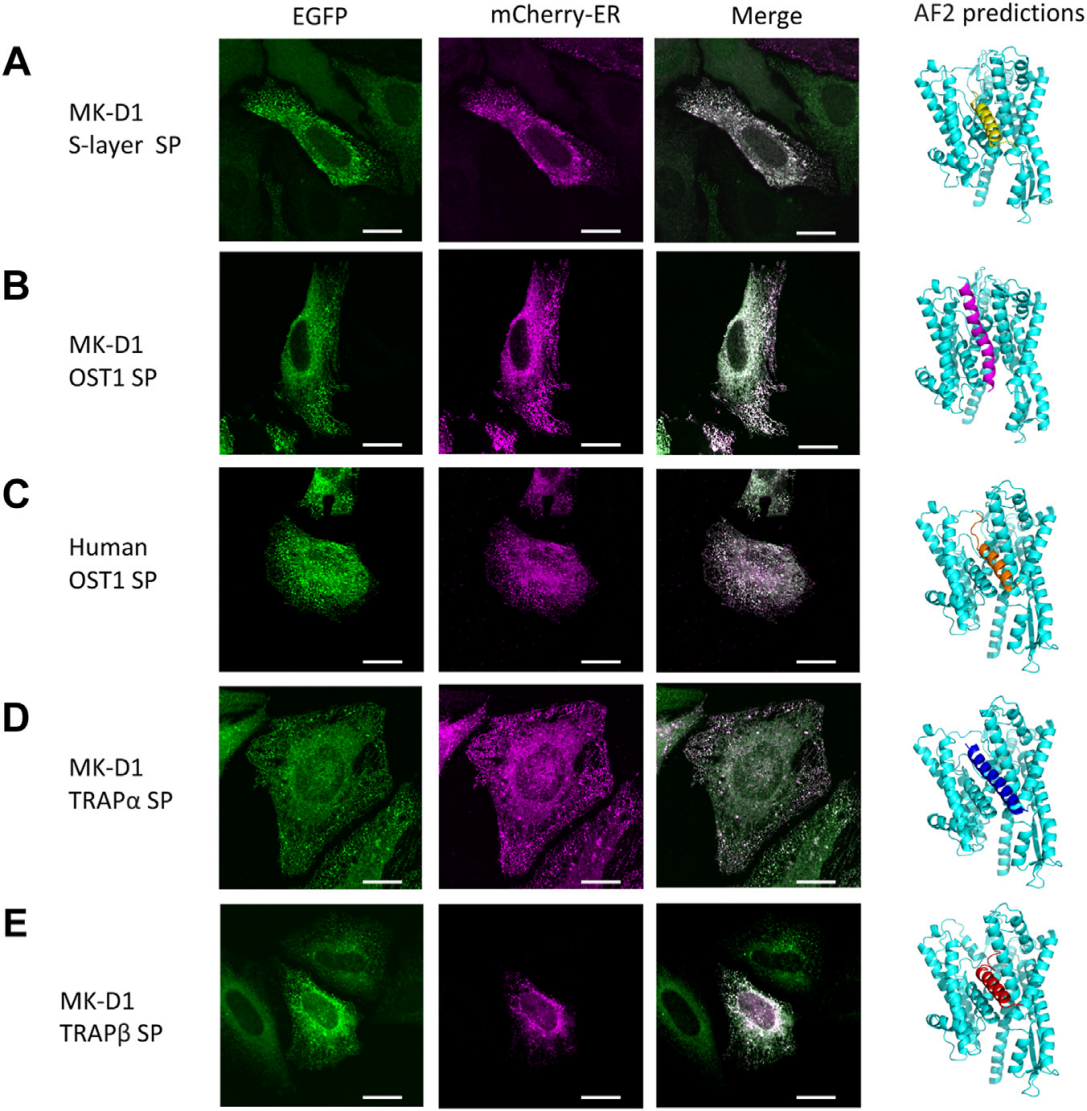
**Representative images of the localization of MK-D1 signal peptide-EGFP chimeras on transfection in HeLa cells.** Signal peptides (SP) alone, from MK-D1 proteins, were fused to EGFP and co-transfected with the ER marker into HeLa cells as in Figure 1. *A*, MK-D1 S-layer protein SP. *B*, MK-D1 OST1 SP. *C*, human OST1 SP control. *D*, MK-D1 TRAPα SP. *E*, MK-D1 TRAPβ SP. AF2 co-predictions are shown as c*artoons* for the human Sec61α and each of the signal peptides. Sec61α, *cyan*; S-layer SP, *yellow*; MK-D1 OST1 SP, *magenta*; Human OST1 SP, *orange*; MK-D1 TRAPα SP, *blue*; MK-D1 TRAPβ SP, *red*. Scale bar = 20 μm. Quantification of the colocalization and distributions relative to the plasma membrane are found in Figs. S3 and S4, and AF2 statistics are given in Fig. S5. AF2, AlphaFold2; ER, endoplasmic reticulum; MK-D1, Candidatus Prometheoarchaeum syntrophicum strain MK-D1; OST, oligosaccharyltransferase; SP, signal peptide; TRAP, translocon-associated protein.

To demonstrate that MK-D1 signal peptides are responsible for ER localization, we expressed a series of signal sequences fused to EGFP in HeLa cells (Fig. 2). The signal peptides from the MK-D1 S-layer protein, TRAPα, TRAPβ, and OST1 all localized to the ER, as did the positive control human OST1 (Fig. 2*C*) and did not show the diffuse expression pattern of EGFP alone (Fig. 1*D*). Thus, the MK-D1 signal peptides are responsible for directing localization to the ER.

To further assess the compatibility of Asgard signal peptides with eukaryotic Sec61α, we used AlphaFold2 (AF2) (35, 36) to predict the complex structures of human Sec61α with the MK-D1 signal peptides (Fig. 2). In each prediction, the MK-D1 signal peptide occupies the lateral gate of human Sec61α in a similar orientation to the structure of a signal peptide-engaged Sec61 complex (37), and also observed in the control human OST1 signal peptide with human Sec61α AF2 prediction (Fig. 2*C*). Taken together, these data indicate that Asgard signal peptides are compatible with the eukaryotic Sec61α translocase, and when heterologously expressed in eukaryotic cells, Asgard signal peptide-containing proteins are directed to the site of eukaryotic Sec61 localization, the ER.

### Processing and glycosylation

**Figure 3:**
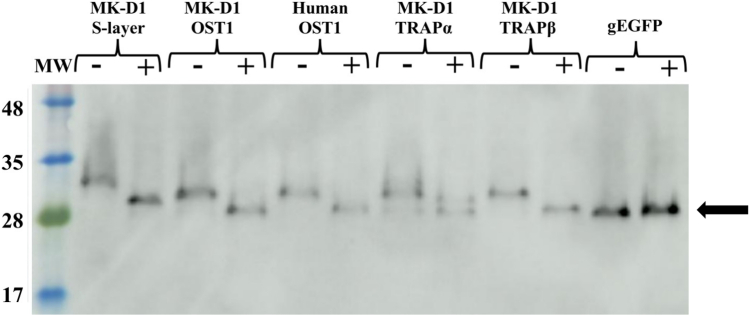
**Glycosylation and processing of the signal peptide-EGFP chimeras with an N-glycosylation acceptor site, on transfection in HeLa cells.** The Western blot was produced from total cell samples probed with an anti-EGFP primary antibody. + and − indicate the cells were grown in the presence or absence of tunicamycin, an N-linked glycosylation inhibitor, respectively. gEGFP refers to EGFP with an N-glycosylation acceptor site but without a signal peptide. This construct is not targeted to the endoplasmic reticulum, and its migration position (size, *black arrow*) is equivalent to the processed non-glycosylated signal peptide-EGFP chimeras. Migration at higher molecular weight positions, relative to gEGFP in the +tunicamycin lanes indicates a lack of cleavage of the signal peptides. Migration at higher molecular weight positions of each chimera in the −tunicamycin lane, relative to the +tunicamycin lane, indicates glycosylation. The full Western blot is shown in Fig. S6. MW, molecular weight markers labeled in kDa. MK-D1, Candidatus Prometheoarchaeum syntrophicum strain MK-D1.

To investigate whether the MK-D1 signal peptides undergo processing and N-glycosylation by the human Sec61/OST/TRAP translocon, we expressed these signal peptides fused to a variant of EGFP containing an N-glycosylation acceptor site (gEGFP, N147T) (38). HeLa cells transfected with these constructs were cultured with or without the N-glycosylation inhibitor tunicamycin (39) and subsequently analyzed by Western blot using an EGFP primary antibody. In control experiments, gEGFP alone showed similar migration patterns with or without the N-glycosylation inhibitor (Fig. 3), suggesting that gEGFP lacking a signal peptide is not glycosylated. Fusion of the human OST1 signal peptide to gEGFP displayed comparable migration in the presence of the N-glycosylation inhibitor, indicative of signal peptide cleavage by Sec61. In the absence of an inhibitor, the band migrated at a higher molecular weight, consistent with glycosylation. Similarly, the fusion of the MK-D1 OST1 and TRAPβ signal peptides to gEGFP exhibited similar patterns to human OST, indicating both signal peptide cleavage and glycosylation. The TRAPα signal peptide fused to gEGFP showed two bands in the presence of tunicamycin, as it did in the absence of an inhibitor, indicating partial signal peptide cleavage and glycosylation. Conversely, the S-layer fusion protein migrated as a higher molecular weight band than gEGFP in the presence of tunicamycin, indicating no cleavage of the predicted signal peptide. In the absence of tunicamycin, a shift to higher molecular weight indicated glycosylation.

This experiment reveals that some MK-D1 signal peptides, including those from OST1 and TRAPβ, are processed efficiently by the human translocon, as evidenced by their ability to be cleaved and, their gEGFP reporter, glycosylated. The MK-D1 TRAPα signal peptide showed incomplete cleavage, indicating partial compatibility. By contrast, the S-layer signal peptide was not cleaved but was glycosylated, suggesting either that the signal peptide cleavage site is incompatible with cleavage, or it was erroneously predicted. Together, these data reveal that MK-D1 signal peptides are targeted to the human translocon, where they are processed at various levels of efficiency.

### Asgard translocon transmembrane proteins

**Figure 4:**
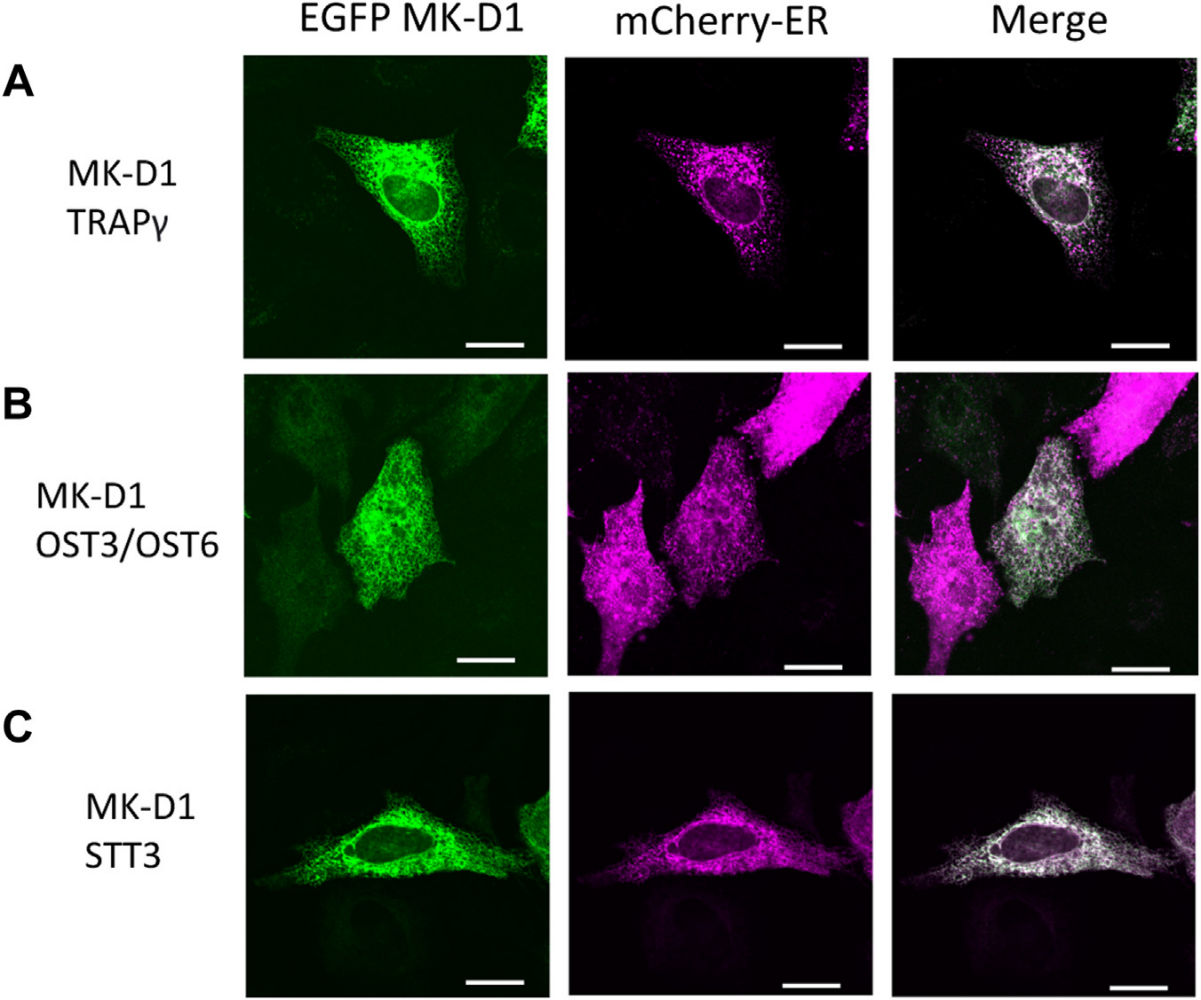
**Representative images of the localization of non-signal peptideMK-D1 transmembrane proteins on transfection in HeLa cells.** HeLa cells were co-transfected to express each of EGFP-fused MK-D1 (*A*) translocon-associated protein γ, (*B*) OST3/OST6, and (*C*) STT3 with the endoplasmic reticulum localizing mCherry construct. At 24 h post-transfection, cells were fixed and imaged with the confocal microscope. Quantification of the colocalization and distributions relative to the plasma membrane are found in Figs. S7, *A*–*C* and S8, *A*–*C*. Scale bar = 20 μm. MK-D1, Candidatus Prometheoarchaeum syntrophicum strain MK-D1; OST, oligosaccharyltransferase.

To determine whether Asgard transmembrane proteins, which lack signal peptides, are preferentially located in eukaryotic cells (Table 1), we expressed this class of proteins from the MK-D1 OST and TRAP translocon complexes as EGFP fusion proteins in HeLa cells, together with the mCherry ER marker. EGFP tagged MK-D1 TRAPγ, OST3/6, and STT3 all colocalized with the ER marker (Fig. 4). Thus, the entire set of MK-D1 OST and TRAP complex subunits are located in the ER when expressed in eukaryotic cells.

**Figure 5:**
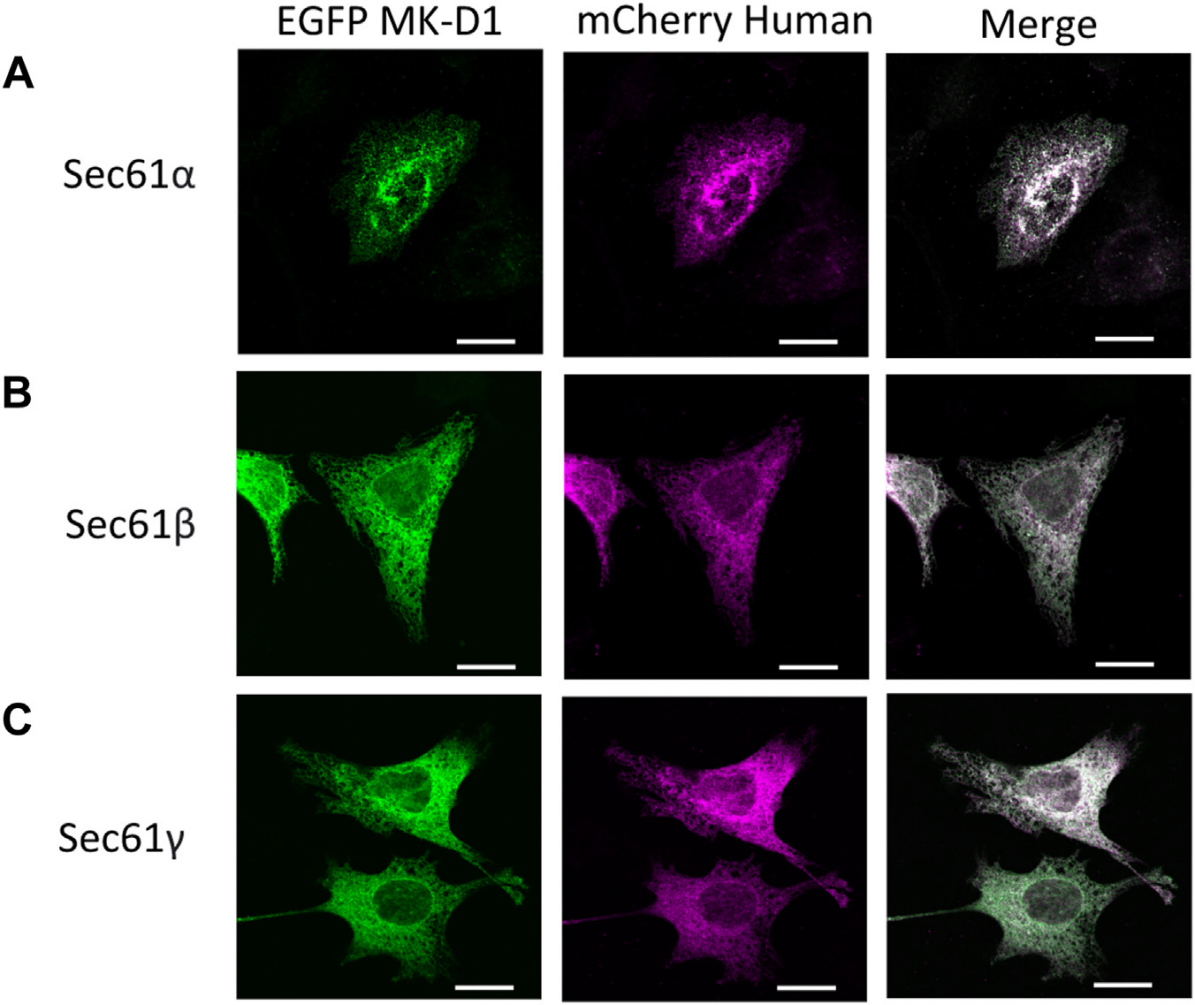
**Representative images of the localization of MK-D1 Sec61 proteins on transfection in HeLa cells.** Each of EGFP-tagged MK-D1 *A*, Sec61α, *B*, Sec61β and *C*, Sec61γ was co-transfected with its corresponding mCherry fused-human counterpart into HeLa cells. Quantification of the colocalization and distributions relative to the plasma membrane are found in Figs. S7, *D*–*F* and S8, *D*–*F*. Scale bar = 20 μm. MK-D1, Candidatus Prometheoarchaeum syntrophicum strain MK-D1.

Next, we co-expressed the three components of the MK-D1 Sec61αβγ preprotein translocase complex as EGFP fusion proteins pairwise with the corresponding human Sec61αβγ subunits mCherry fusion proteins (Fig. 5). In each case, the MK-D1 and human EGFP and mCherry fluorescence colocalized, indicating that the MK-D1 Sec61 is directed to the same compartment as human Sec61, the ER.

**Figure 6:**
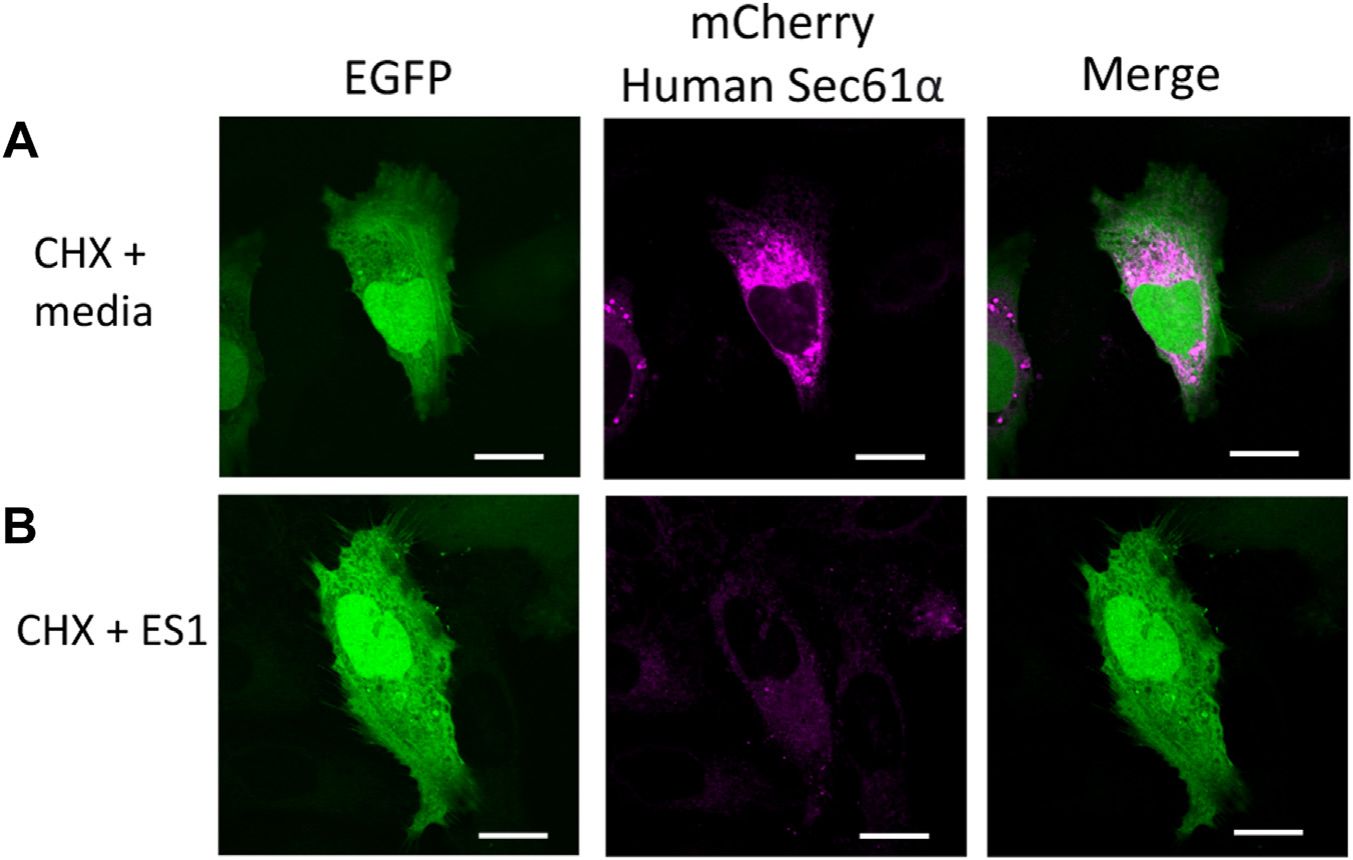
**Representative images of the effect of Sec61 inhibition on the localization ofCandidatus Prometheoarchaeum syntrophicum strain MK-D1newly synthesized Sec61 proteins in HeLa cells.***A*–*C*, HeLa cells were co-transfected with EGFP and mCherry fused human Sec61α. At 20 h post-transfection, cycloheximide (CHX) was added to a final concentration of 100 μg/ml for 3 h, followed by (*A*) the addition of media or (*B*) the addition of Eeyarestatin 1 for 3 h before imaging. Scale bar = 20 μm.

Finally, we asked whether the presence of Sec61α in the ER mediates the insertion of new Sec61α. To do this, HeLa cells were co-transfected with EGFP and mCherry fused human Sec61α. At 20 h post-transfection, cycloheximide (CHX) was added to halt protein translation in the ribosomes. After 3 h the media was reverted to CHX-free media to allow translation either in the absence or presence of eeyarestatin 1, a Sec61α inhibitor (40, 41). After 3 h, cells not treated with the Sec61α inhibitor exhibited robust Sec61α expression (Fig. 6*A*), while cells incubated with the Sec61α inhibitor showed little Sec61α expression (Fig. 6*B*). Barring off-target effects, these findings indicate that translation and insertion of new Sec61α molecules require the existence of a functional Sec61α molecule for ribosome docking, translation, and translocation.

Taken together, these data establish the principle that the location of the existing Sec61 complex, which engages the ribosome, determines the site at which the new Sec61/OST/TRAP translocons are located. The co-translational insertion of Sec61α is dependent on the existence of a functional Sec61α molecule. Thus, the translocon location is inherited at the protein molecular level rather than at the genetic level, which likely has implications for ER maintenance, disease, and, in our focus, evolution.

### Asgard translocon structure and ribosome interaction

**Figure 7:**
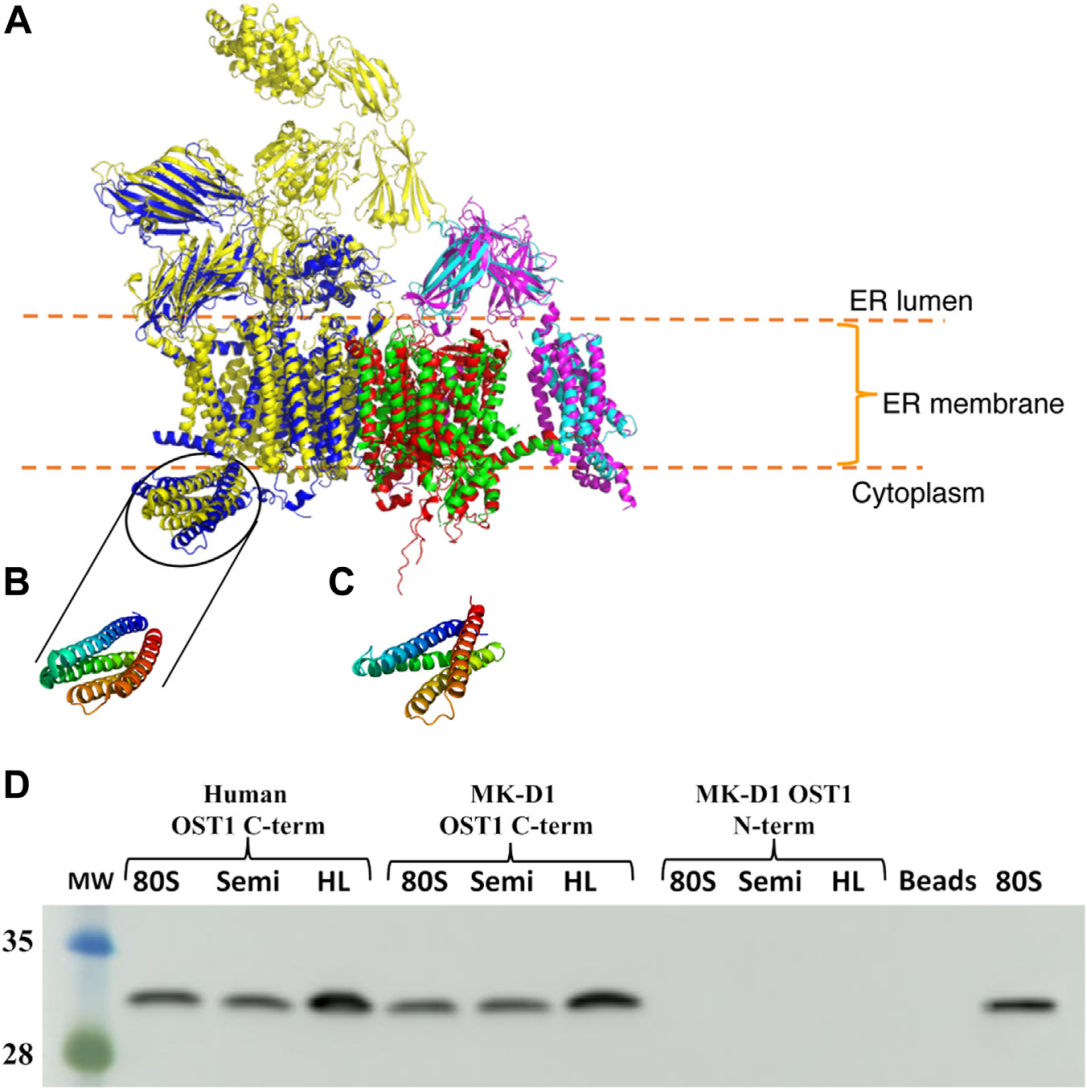
**The structural and functional relationship between the MK-D1 and human translocons.***A*, AF2 predicted models for MK-D1 Sec61 (*red*), OST (*blue*), and TRAP (*cyan*) complexes superimposed onto the human Sec61 (*green*), OST (*yellow*), and TRAP (*pink*) translocon structure (18) (PDB 8B6L). AF2 statistics are given in Fig. S9. *B*, structure of the human OST1 cytosolic domain (ribosome interacting domain). *C*, a 1.85 Å X-ray crystal structure of the cytosolic domain of MK-D1 OST1. *D*, pull-down assay showing interactions of His-tagged versions of the cytosolic domain of MK-D1 OST1(C-term), cytosolic domain of human OST1 (C-term), and the extracellular domain of MK-D1 OST1 (N-term) with various preparations of ribosomes. Beads, control experiment without His-tagged protein, and the final lane the 80S standard. The Western blot is probed with an antibody against ribosomal protein S3 (RPS3). The full Western blot is shown in Fig. S10. The quality of the 80S ribosomes is shown in Fig. S11. 80S, highly purified ribosomes; HL, HeLa cell clarified lysate; MW, molecular weight markers labeled in kDa; Semi, semi-purified ribosomes. AF2, AlphaFold2; MK-D1, Candidatus Prometheoarchaeum syntrophicum strain MK-D1; OST, oligosaccharyltransferase; TRAP, translocon-associated protein.

To probe the global structural similarities between the MK-D1 and eukaryotic Sec61/OST/TRAP translocons, we constructed the AF2 predicted models (35, 36) of MK-D1 Sec61, OST and TRAP complexes and superimposed them onto the eukaryotic Sec61/OST/TRAP translocon cryo-electron microscopy (cryoEM) structure (18). The Asgard model and human translocon structure show significant structural homology (Fig. 7*A*). We were particularly intrigued by the cytoplasmic C-terminal domain of OST1, which forms a cytoplasmic helical bundle in the MK-D1 model and in the eukaryotic structure (Fig. 7*A*, circled). To confirm the validity of the AF2 model in this region, we expressed, purified, crystallized, and solved the X-ray structure of this domain (Table 2). The MK-D1 OST1 domain forms a 4-helix bundle with the same topology as the human domain (Fig. 7, *B* and *C*), although the helices have slightly different angles relative to each other.

**Table 2:** X-ray data collection and refinement statistics

	MK-D1 OST1 cytoplasmic domain WP_147663064.1 (PDB code 8WHN)
Crystals	
Crystallization conditions	0.1 M Bis-Tris propane, pH 6.5 18% PEG 3350 0.3 M NaF
Lattice	P22_1_2_1_
*a*, *b*, *c* (Å)	41.4, 58.7, 67.6
*α*, *β*, *γ* (°)	90.0, 90.0, 90.0
Data collection	
Beamline	BL41XU, SPring-8
Wavelength (Å)	1.0
Resolution (Å)	20.0–1.95 (2.00–1.95)
*R*_merge_	10.6 (158.5)
*R*_meas_	11.6 (171.9)
*R*_pim_	4.7 (66.0)
*I*/σ(*I*)	8.0 (1.3)
*CC*_1/2_	(0.783)
Completeness (%)	99.7 (99.9)
Redundancy	6.3 (6.6)
Refinement	
Resolution (Å)	20.0–1.95 (2.02–1.95)
No. reflections	12,432 (1230)
*R*_work_/*R*_free_	21.8/24.6 (40.6/41.1)
No. atoms	
Protein	1258
Water	39
*B* factors	
Protein	62.1
Water	54.3
r.m.s deviations	
Bond lengths (Å)	0.006
Bond angles (°)	0.76
Ramachandran Plot	
Favoured (%)	98.6
Outliers (%)	0.0

Abbreviation: MK-D1, Candidatus Prometheoarchaeum syntrophicum strain MK-D1; OST, oligosaccharyltransferase.

Finally, to ascertain whether this cytoplasmic domain of MK-D1 OST1 can interact with ribosomes, we produced histidine-tagged domains from MK-D1 and human OST1 and carried out pulldowns on nickel-nitrilotriacetic acid (NTA) beads against HeLa cell lysate, and semi-purified and highly purified human ribosomes. Western blot analysis using a ribosome-specific antibody was used to probe the interaction with ribosomes. Ribosomes were detected to bind to the human and MK-D1 cytoplasmic C-terminal domains of OST1, but not to the negative control, the MK-D1 OST1 extracellular N-terminal domains (Fig. 7*D*). The C-terminal domains of OST1 were able to recruit ribosomes from samples with different levels of ribosome purity, indicating a direct and specific interaction. This finding suggests that MK-D1 OST1 participates in the recruitment of MK-D1 ribosomes to the Sec61/OST/TRAP translocon at the cell membrane for translation and translocation of membrane proteins and preproteins. A comparison of the surface residues of the human and MK-D1 cytoplasmic C-terminal domains of OST1 reveals surface patches of basic residues that cluster in regions that may be able to interact with the RNA components of the ribosome (Fig. S12). Taken together, these data demonstrate that the Asgard and eukaryotic Sec61/OST/TRAP translocon machinery are structurally and functionally similar and show a level of mutual compatibility. Namely, the eukaryotic ribosome-translocon complex can translate, process, and direct Asgard translocon proteins to the ER, and the Asgard translocon complex can interact with eukaryotic ribosomes.

## Discussion

It is known that the eukaryotic translocon is compatible with preproteins from diverse organisms. For instance, bacterial β-lactamase and periplasmic maltooligosaccharide binding protein (MalE) can be secreted from eukaryotic cells, *via* the ER, using their native signal sequences (42, 43), and bacterial membrane proteins can be produced in mammalian cells (44). Here, we have extended this principle to demonstrate that all components of the MK-D1 Sec61/OST/TRAP translocon are directed to the ER. Similarly, it is known that in archaea ribosomes are located to the cell membranes (45, 46, 47) and bind directly to SecY/Sec61 translocon (45). Here, we show that the cytoplasmic C-terminal domain of archaea OST1 also mediates interactions with the ribosome, and specifically, the MK-D1 OST1 complex can engage human ribosomes, despite the ∼1.8 BY of divergence of the two species (27). While the question of Asgard translocon functional activity will drive our future research, it is clear from the fluorescence images of the hybrid transmembrane GFP constructs that GFP is functional and that the MK-D1 Sec61/OST/TRAP translocon subunits are directed to the ER. The mechanisms for MK-D1 protein retention in the ER are unknown. Interactions with the native translocon components or quality control systems acting on misfolded proteins (48) may both play roles. Nevertheless, the compatibility of parts of the Asgard and eukaryotic ribosome-translocon systems, demonstrated here, has implications for the emergence of the ER during evolution.

### Implications for eukaryogenesis

**Figure 8:**
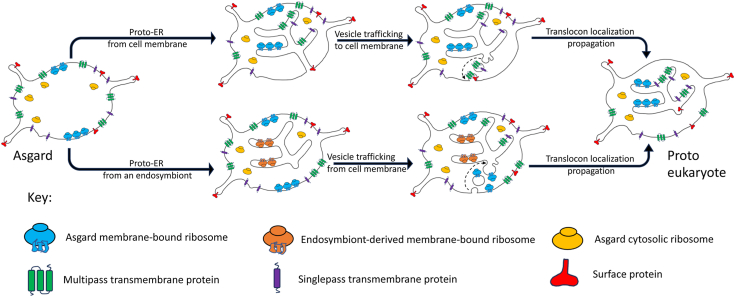
**Hypothetical models of ER biogenesis mediated by translocon-location propagation.***Top route* from models where the proto-ER arises from Asgard cell membrane invagination or expansion. *Bottom route* from models where the proto-ER arises from an endosymbiont residing within an Asgard cell. ER, endoplasmic reticulum.

Asgard and eukaryotic Sec61/OST/TRAP compatibility is consistent with models of eukaryogenesis in which the eukaryotic cell membrane and cytoplasm are derived from an Asgard archaeon (49). Furthermore, the proto-ER membrane should be derived from a membrane that allows docking of Asgard cytoplasmic ribosomes onto the translocon in the cell membrane and proto-ER. Invagination or cell expansion models leading to the emergence of the proto-ER from the cell membrane are consistent with this scenario (Fig. 8). However, such models require the additional steps of the emergence of vesicle budding and fusion at the cell surface to complete the transport of transmembrane proteins from the proto-ER to the cell membrane. Models of ER origin that involve membranes from an endosymbiont residing within the Asgard archaeon have an initial problem in that the translocons are inverted and cannot engage Asgard cytoplasmic ribosomes (Fig. 8). However, the emergence of early endocytic vesicle trafficking may have allowed for the shuttling of Asgard translocons, in the correct orientation, from the cell surface to the proto-ER.

The potential sources of the proto-ER membrane require either the emergence of endocytic or exocytic vesicle transport. These processes occur in extant eukaryotes, and many homologs of eukaryotic-like vesicle transport proteins have been identified in Asgard archaea (30), suggesting that the emergence of vesicle transport is a realistic step within an Asgard-derived cytoplasm. However, the lack of chaperones containing signal peptides in Asgard sequence databases suggests that a proto-ER, in which chaperone-assisted refolding occurs, is not present in the extant Asgard organisms sequenced to date.

Here we have shown that translocon inheritance occurs at three levels in HeLa cells. Sec61 is encoded at the genome level, and Sec61 processing and location are inherited at the protein and organelle levels, respectively. We hypothesize that this mechanism, by which the existing translocon location directs future translocon distribution (translocon-location propagation), will apply to the proto-eukaryote. During the emergence of the proto-ER, translocons likely co-existed on the cell and proto-ER membranes (Fig. 8), providing an opportunity for the proto-ER to evolve efficient protein modification and folding, and vesicle transport of transmembrane proteins. We suggest that once the quality of folded and modified transmembrane proteins from the proto-ER surpassed that of proteins made at the cell membrane, and the vesicle transport system became efficient, then positive selection combined with translocon-location propagation would have relocated the entire population of translocons to the proto-ER (Fig. 8).

## Experimental procedures

### Protein expression and purification

The NCBI accession codes of the protein sequences can be found in Table S1. The cytosolic (C-terminal) domains of MK-D1 OST1 (residues 449–607) and human OST1 (residues 465–607) and the extracellular (N-terminal) domains of MK-D1 OST1 (residues 33–425) were codon optimized for expression in *Escherichia coli*, synthesized (GeneScript) and placed in the pSY5 vector (50), and expressed in BL-21 *E. coli*. The proteins were affinity purified on Ni-NTA resin (FUJIFILM Wako Chemicals), cleaved with HRV 3C protease and further purified by gel chromatography (Bio-Rad) by standard protocols (51). The proteins were exchanged to the crystallization buffer (10 mM Tris-HCl, pH 7.5, 30 mM NaCl) and concentrated to 10 mg/ml protein with 10 kDa MWCO centrifuge filters (Merck).

### Site-directed mutagenesis and selenomethionine incorporation

PCR-based site-directed mutagenesis was used to introduce mutations at position 532 (Ile532Met) of the cytosolic domain of OST1 using QuikChange site-directed mutagenesis. Selenomethionine was incorporated into this variant in M9 medium (1X M9 salt solution, 1 mM MgSO_4_, CaCl_2_, 0.4% Glucose, 1X BME Vitamin) supplemented with lysine, threonine, isoleucine, leucine, valine, and phenylalanine.

### Crystallization, structure determination, model building, and refinement

Crystallization screening of purified MK-D1 C-terminal OST1 was performed by sitting-drop vapor diffusion method. Optimized crystals were grown from 0.1 M Bis-Tris propane, pH 6.5, 18% PEG 3350, 0.3 M NaF. Protein crystals were harvested, and flash frozen in liquid nitrogen for X-ray diffraction. X-ray data were collected on BL41XU (λ = 1.0 Å) SPring-8 on a Pilatus 6M detector. The selenomethionine-grown protein crystals diffracted to superior resolution, 1.95 Å. No appreciable selenium signal was detected indicating that these proteins did not have a significant amount of incorporated selenomethionine. Data were indexed, scaled, and merged following standard protocols (51). Molecular replacement and refinement were carried out using an AlphaFold2-generated model (35).

### Human ribosome purification

80S ribosomes were purified from HeLa cells cultured in MEM media. Briefly, HeLa cells in ten 15 cm diameter petri dishes at 70 to 80% confluency were washed three times with 5 ml cold PBS (pH 7.4) and scraped off in 1 ml cold Buffer A (20 mM HEPES, pH 7.4, 100 mM KOAc, 7.5 mM Mg(OAc)_2_). The cells were ruptured using a needle with repeated suction and release (30 times). The cell lysate was centrifuged at 20,000*g* for 10 min at 4 °C. The supernatant was gently layered on top of a 10 to 40% sucrose density gradient in buffer A and centrifuged at 28,000 rpm for 4.5 h at 4 °C using the SW28 rotor (Beckman Coulter). The gradients were fractionated from the top to the bottom using a Gradient Master (BioComp). The fractions corresponding to the 80S peak (Fig. S11*A*) were collected, and concentrated using a 100 kDa Amicon concentrator and the concentration was measured at A_260nm_. The pure 80S human ribosomes were confirmed by imaging of negative stained samples by electron microscopy (52) (Fig. S11*B*).

Semi-purified human ribosomes were prepared by an alternate protocol (53). Briefly, cells were harvested in 10 ml cold DPBS pH 7.4, and centrifuged for 5 min at 500*g*, 4 °C. Cell pellets were sequentially resuspended in three 100 μl-additions of Buffer B (250 mM sucrose, 250 mM KCl, 5 mM MgCl_2_, 50 mM Tris-HCl, pH 7.4). The cell suspension was then treated with NP-40 detergent to a final concentration of 0.7%. Detergent-treated cells in Buffer B were incubated on ice for 10 to 15 min with gentle pipetting at 5 min intervals and then centrifuged at 750*g* for 10 min at 4 °C. The supernatant fraction was clarified by centrifugation at 12,500*g* for 10 min at 4 °C. 4 M KCl solution was added to give a final concentration of 0.5 M KCl. The KCl-adjusted supernatant was layered over a 1 ml sucrose cushion (1 M sucrose, 0.5 M KCl, 5 mM MgCl_2_, 50 mM Tris-HCl, pH 7.4) in a 3 ml polycarbonate tube. This was balanced with buffer C (250 mM sucrose, 0.5 M KCl, 5 mM MgCl_2_, 50 mM Tris-HCl, pH 7.4) and centrifuged at 250,000*g* for 2 h at 4 °C in an ultracentrifuge. The translucent pellet was rinsed twice with 200 μl cold water and resuspended with three 100 μl additions of buffer D (25 mM KCl, 5 mM MgCl_2_, 50 mM Tris-HCl, pH 7.4).

Cells extracts for pulldowns were prepared from HeLa cells cultured to 70 to 80% confluency in 15 cm petri dishes and washed three times with cold PBS (pH 7.4). The cells were scraped off in 100 μl lysis buffer (20 mM HEPES, pH 7.4, 100 mM KOAc, 7.5 mM Mg(OAc)_2,_ 1% NP-40) into 1.5 ml tubes and allowed to rotate gently for 30 min on a TAITEC Rotator RT-5 at 4 °C. The cell lysate was centrifuged at 14,000 rpm for 10 min, and 100 μl of the supernatant was used for pull-down assays.

### Western blotting

His-tagged versions of the C-terminal (cytosolic) domains of MK-D1 OST1 and human OST1 and the extracellular N-terminal domains of MK-D1 OST1 were affinities purified on Ni-NTA agarose columns (FUJIFILM Wako Chemicals). A tag-based pull-down assay on Ni-sepharose His SpinTrap columns (Cytiva) was used to probe for interactions between the purified ribosomes and His-tagged versions of the cytosolic domain of MK-D1 OST1, cytosolic domain of human OST1, and N-terminal domain of MK-D1 OST1. Briefly, the Ni-sepharose column was first equilibrated with binding buffer A (20 mM HEPES, pH 7.4, 100 mM KOAc, 7.5 mM Mg(OAc)_2_). The Ni-sepharose column was mixed with 50 μl of 8 mg/ml of each affinity-purified His-tagged protein in binding buffer and allowed to stand for 5 min. A column not bound to His-tagged protein was also used as a negative control (beads). The Ni-sepharose resin was washed three times with 800 μl of binding buffer. Next, 50 μl of 80 nM pure human 80S ribosomes, 50 μl of semi-purified (semi) ribosomes (A_260 nm_ 8 mg/ml), and 100 μl of HeLa lysate were added to their respective columns and washed three times with 800 μl of binding buffer. Elution was performed with 400 μl of elution buffer (20 mM HEPES, pH 7.4, 100 mM KOAc, 7.5 mM Mg(OAc)_2_, 250 mM imidazole). Eluted fractions representing bait-prey complexes were concentrated to 100 μl, and 15 μl aliquots from each sample were subjected to Western blot analysis probed with an anti-ribosomal protein S3 antibody (RPS3, Cell Signaling Technology, Inc). 15 μl of 30 nM pure 80S human ribosomes was included in the Western blot as a positive control. The Western blot was visualized using the Amersham Imager 680 (Cytiva).

### Cell culture and imaging

HeLa cells were grown in Minimum Essential Media (MEM, Sigma-Aldrich) supplemented with L-glutamine and 10% fetal bovine serum (FBS) (Nichirei), and incubated at 37 °C, 5% CO_2_. *Mycoplasma* contamination in cell cultures was routinely tested using the PCR *mycoplasma* detection set (Takara Bio). At approximately 70% confluence, HeLa cells were co-transfected to express each of EGFP-fused MK-D1 proteins with an endoplasmic reticulum (ER) localizing Sec61β mCherry construct (54), or a plasma membrane marker (mCherry-tagged FERM domain of Ezrin), using the Xfect transfection reagent (Takara Bio). At 24 h post-transfection, cells were washed with PBS (pH 7.4), fixed with 4% paraformaldehyde (Nacalai Tesque, Inc) in PBS for 15 min at room temperature, mounted with Fluoro-KEEPER antifade reagent with DAPI (Nacalai Tesque, Inc), and imaged using FluoView FV1200 confocal microscope (Olympus).

### Quantification of co-localization

A 10.3 × 10.3 μm section from each set of images (EGFP and mCherry channels) was cropped, merged, and used for colocalization analysis in Image J. The plugin, colocalization finder, was used to generate the Pearson correlation coefficient, R, of the colocalization after merging the cropped sections.

### Sec61 inhibition assay

HeLa cells grown to a confluency of approximately 70% were co-transfected with mCherry human Sec61 and EGFP using the Xfect transfection reagent (Takara Bio). At 20 h post-transfection, cycloheximide (CHX) was added to a final concentration of 100 μg/ml for 3 h at 37 °C to halt protein synthesis. The cycloheximide (CHX)-treated media was washed thrice with 1 ml PBS (pH 7.4) within 1 min. The media was exchanged to CHX-free media to allow expression either in the absence or presence of 8 μM Eeyarestatin 1 (55) at 37 °C. Cells were washed, fixed, and imaged as described above after 3 h.

### Glycosylation assay

MK-D1 signal peptides were fused to EGFP bearing an N-glycosylation acceptor site (N147T) (38). HeLa cells at 70% confluence were transfected with DNA encoding the N-glycosylation reporter and treated with or without 1 mg/ml tunicamycin at 9 h post-transfection. Similarly, HeLa cells were transfected with the N-glycosylation reporter without signal peptide, treated with and without tunicamycin were used as controls. At 24 h post-transfection, the cells were placed on ice for 10 min and washed three times with cold PBS (pH 7.4). The cells were scraped off with 100 μl of 1× Laemmli sample buffer and boiled at 95 °C for 5 min. The samples were cooled and 15 μl of each sample was used for SDS-PAGE. Western blot analysis was performed with anti-EGFP primary antibody (Cell Signaling Technology) and visualized with Amersham imager 680 (Cytiva).

### Statistical information

The protein localization experiments were repeated twice. Pull-down and Western blots were performed twice.

## Data availability

The atomic coordinates and structural factor data have been deposited in the PDB database under the accession code 8WHN.

## Supporting information

This article contains supporting information.

## Conflict of interest

The authors declare that they have no conflict of interest with the contents of this article.
