# Supplementary material for: Intercompatibility of eukaryotic and Asgard archaea ribosome-translocon machineries
Source: J Biol Chem. 2024 Aug 14;300(9):107673. doi: 10.1016/j.jbc.2024.107673 (PMC11417166; doi:10.1016/j.jbc.2024.107673)
Supplement: Supplemental Figures and Tables [file mmc1.pdf]

Supporting Information

for

Inter-compatibility of eukaryotic and Asgard archaea translocon complexes

Isaac Carilo[1], Yosuke Senju[1] & Takeshi Yokoyama[2], Robert C Robinson[1,3]

[1]Research Institute for Interdisciplinary Science (RIIS), Okayama University, 3-1-1 Tsushimanaka, Kita-ku, Okayama-shi, Okayama 700-8530, Japan

[2]Graduate School of Life Sciences, Tohoku University, Sendai, Japan

[3]School of Biomolecular Science and Engineering (BSE), Vidyasirimedhi Institute of Science and Technology (VISTEC), Payupnai, Wangchan, Rayong 21210 Thailand

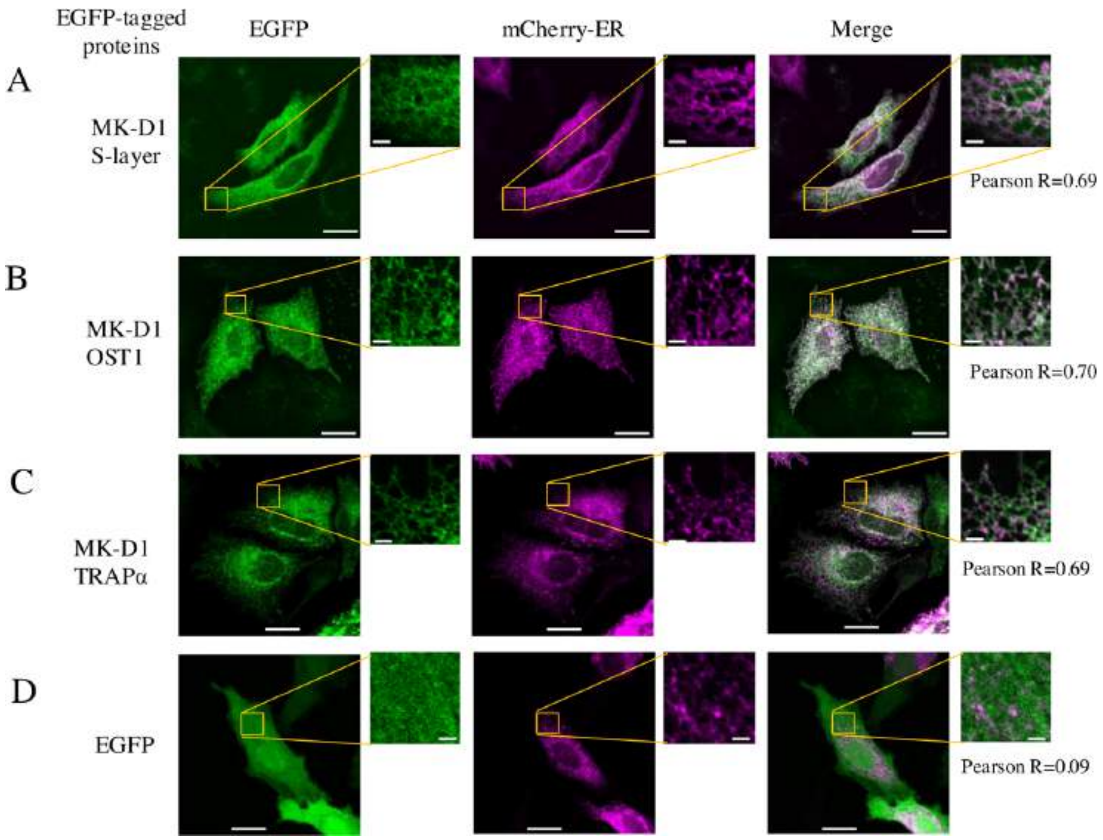

Fig. S1: Pearson correlation evaluation of the colocalization of EGFP-tagged proteins with the mCherry-ER marker from Fig. 1. *A*, S-layer, *B*, OST1 and *C*, TRAPα EGFP-tagged MK-D1 proteins show positive correlation (R = 0.69-0.70) indicating that these proteins associate with the ER. *D*, EGFP shows correlation close to zero (R = 0.09) indicating no directed association with the ER. Scale bar = 20 μm. Scale bar = 2.3 μm in the magnified images.

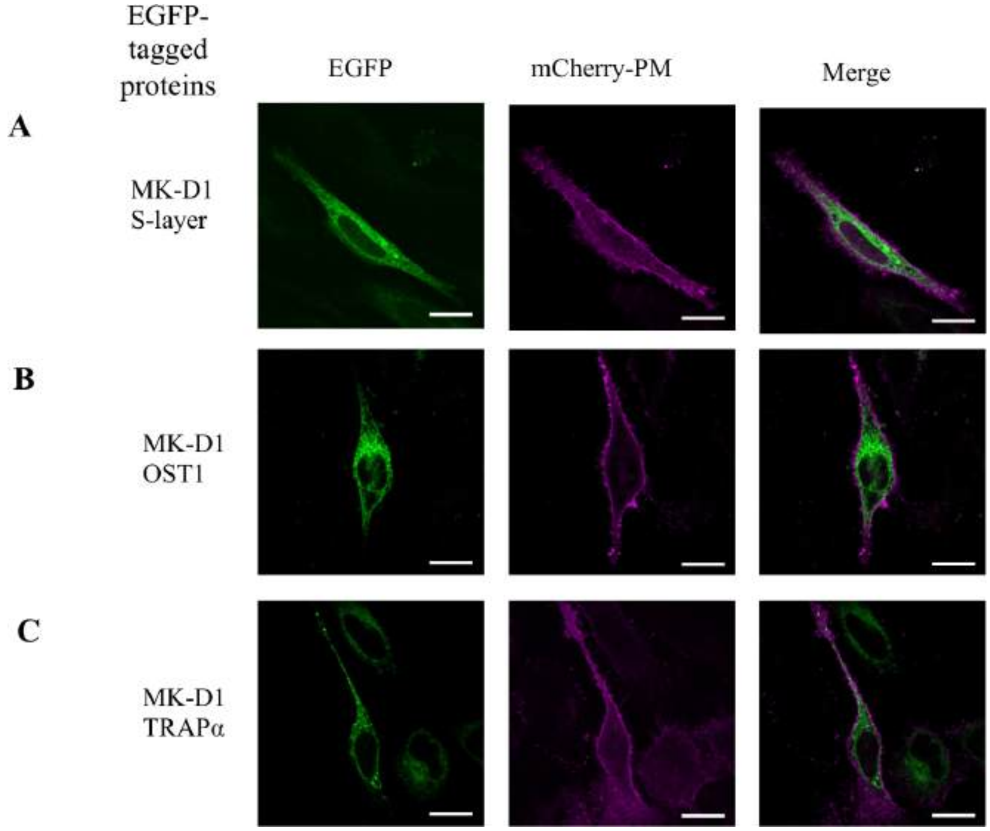

Fig. S2: Colocalization of EGFP-tagged proteins with the mCherry plasma membrane marker as a control for Fig. 1. *A*, S-layer, *B*, OST1 and *C*, TRAPα MK-D1 EGFP-tagged proteins fluorescence (EGFP, green) fall inside the plasma membrane marker (mCherry-PM, magenta) indicating that these proteins are not associated with the plasma membrane. Scale bar = 20 μm.

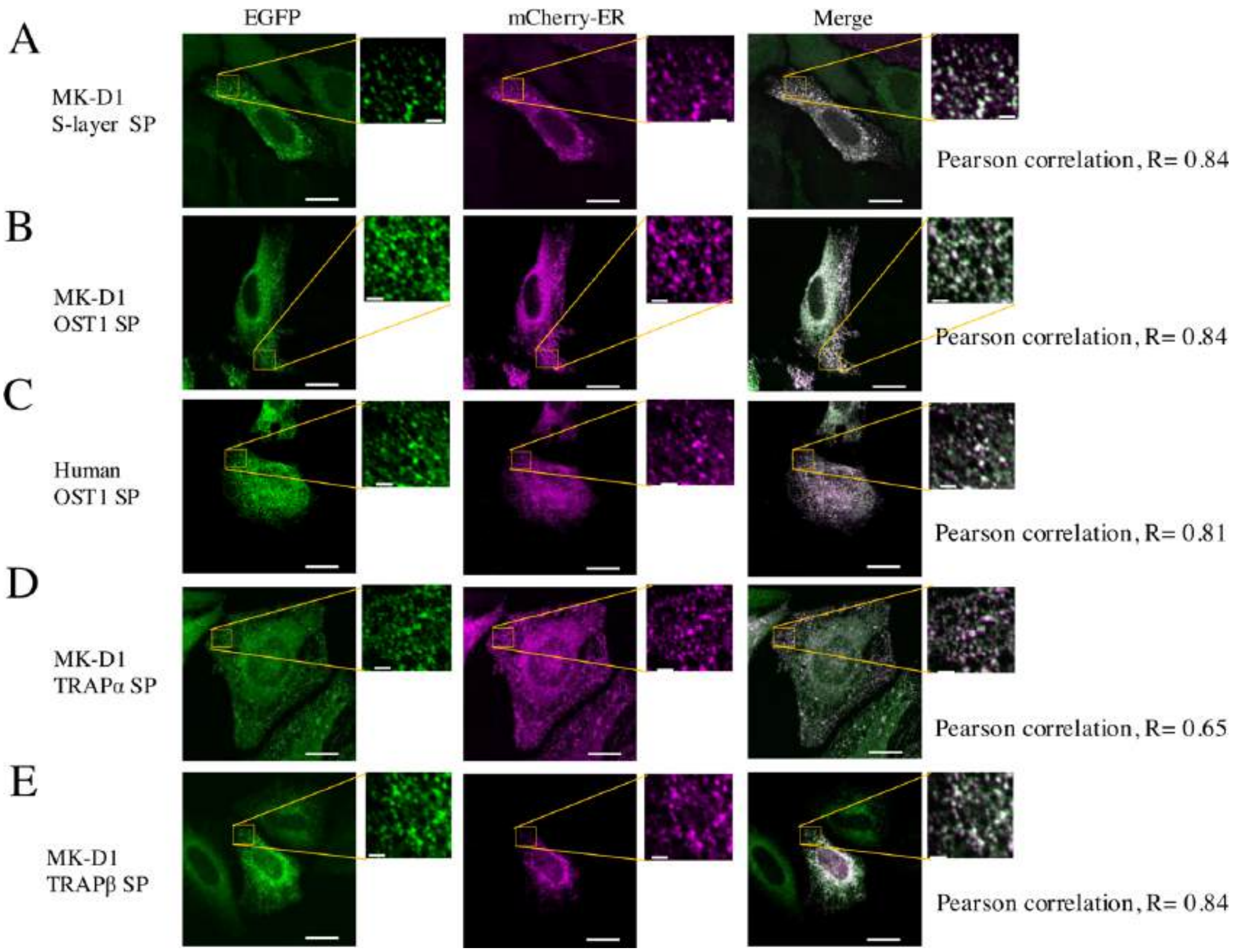

Fig. S3: Pearson correlation evaluation of the colocalization of EGFP-tagged signal peptides (SP) with the mCherry-ER marker from Fig. 2. *A*, MK-D1 S-layer protein SP. *B*, MK-D1 OST1 SP. *C*, Human OST1 SP control. *D*, MK-D1 TRAPα SP. *E*, MK-D1 TRAPβ SP. All 5 signal peptides drive the EGFP to the ER characterized by positive Pearson correlation coefficients (R = 0.65-0.84). Scale bar = 20 µm. Scale bar = 2.3 µm in the magnified images.

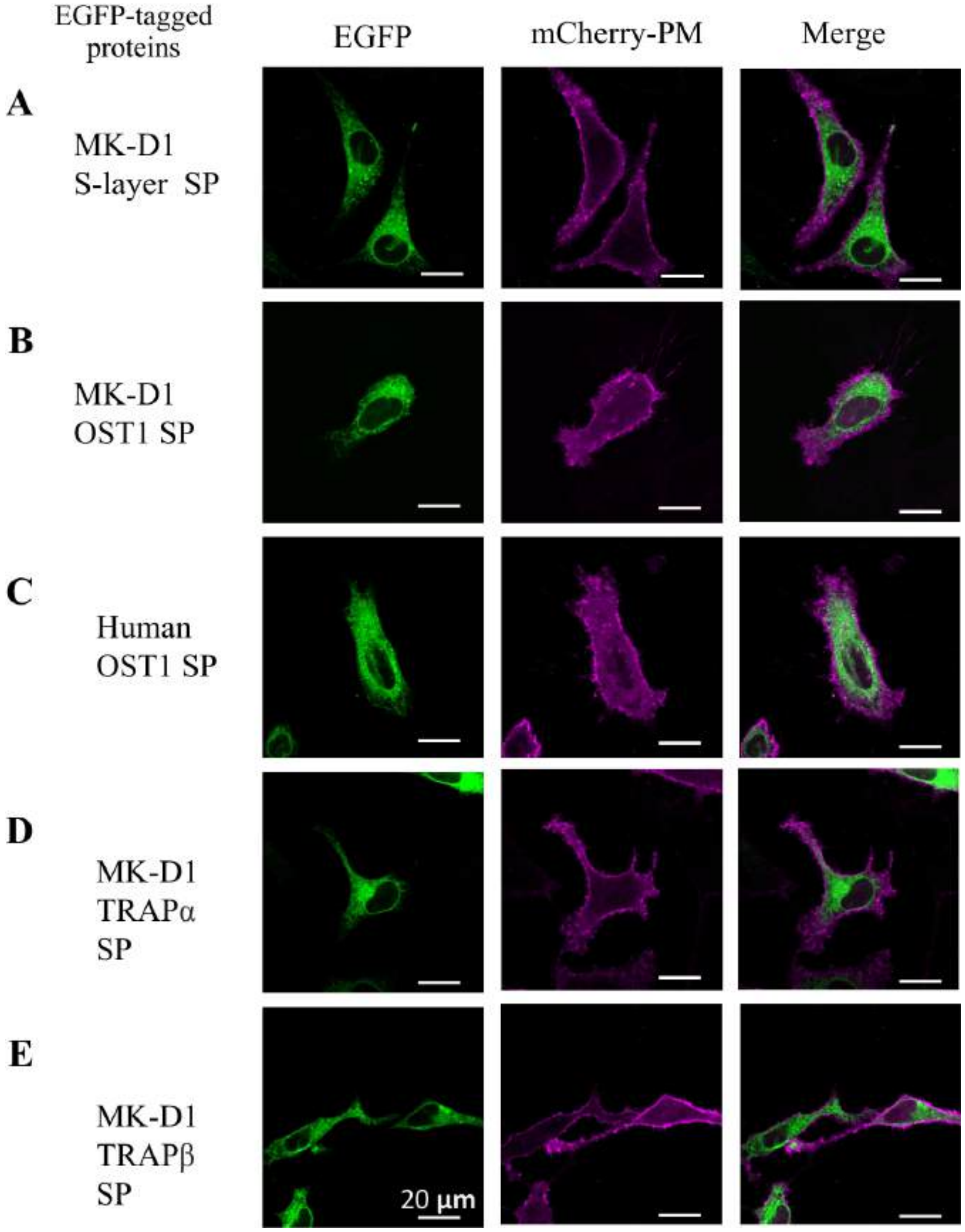

Fig. S4: Colocalization of EGFP from the signal peptides (SP) tagged EGFP constructs with the mCherry plasma membrane marker as a control for Fig. 2. *A*, MK-D1 S-layer protein SP; *B*, MK-D1 OST1 SP; *C*, human OST1 SP control; *D*, MK-D1 TRAPα SP; and *E*, MK-D1 TRAPβ SP EGFP-tagged proteins fluorescence (EGFP, green) fall inside the plasma membrane marker (mCherry-PM, magenta), indicating that these proteins are not associated with the plasma membrane. Scale bar = 20 μm.

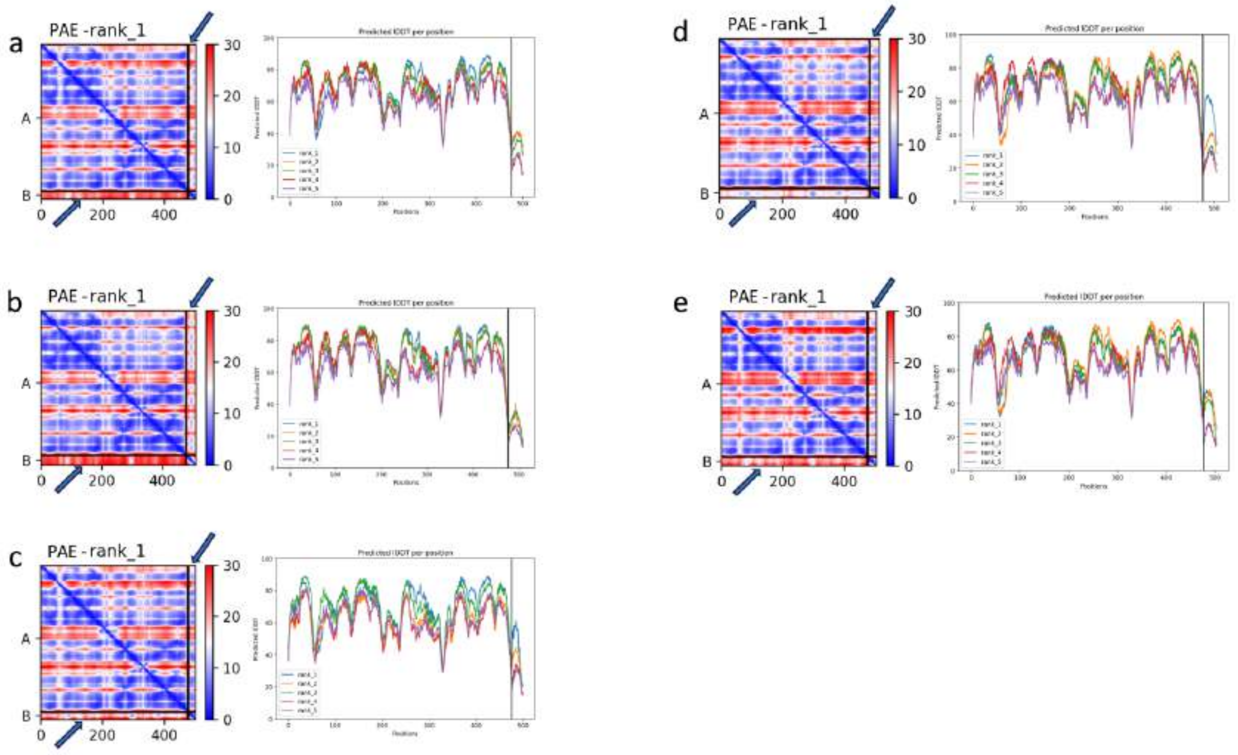

Fig. S5: AF2 statistics for the structure predictions in Fig. 2. AlphaFold2 co-predictions of human Sec61α with the signal peptides of *A*, MK-D1 S-layer, *B*, MK-D1 OST1, *C*, Human OST1, *D*, MK-D1 TRAPα, *E*, MK-D1 TRAPβ. The intermolecular interactions with the signal peptides are highlighted by blue arrows in the PAE plots.

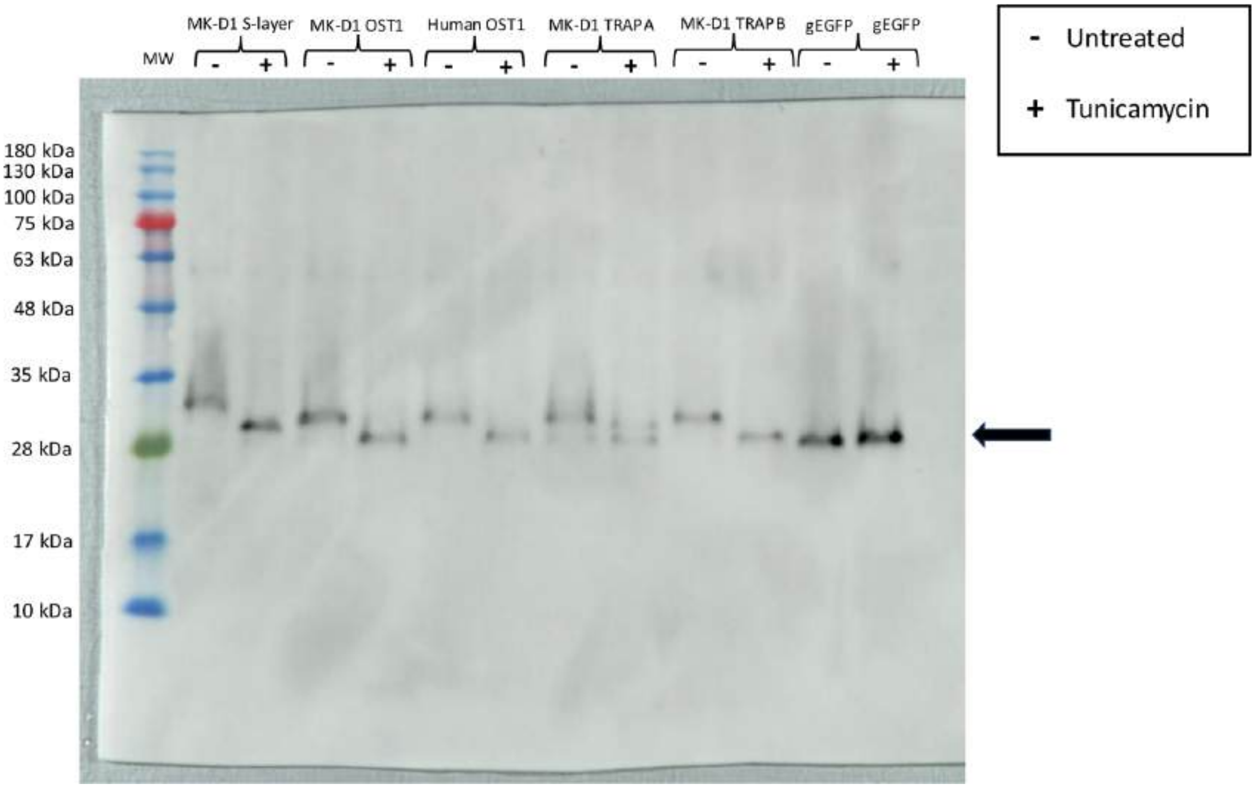

Fig. S6: The entire Western blot for the data shown in Fig. 3. The Western blot was produced from total cell samples probed with an anti-EGFP primary antibody. + and – indicate the cells were grown in the presence or absence of tunicamycin, an N-linked glycosylation inhibitor, respectively. gEGFP refers to EGFP with an N-glycosylation acceptor site but without a signal peptide. This construct is not targeted to the ER, and its migration position (size, black arrow) is equivalent to the processed, non-glycosylated signal peptide-EGFP chimeras. Migration at higher molecular weight positions, relative to gEGFP in the + tunicamycin lanes indicate lack of cleavage of the signal peptides. Migration at higher molecular weight positions of each chimera in the - tunicamycin lane, relative to the + tunicamycin lane, indicates glycosylation. The full Western blot is shown in Fig. S6. MW, molecular weight markers labelled in kDa.

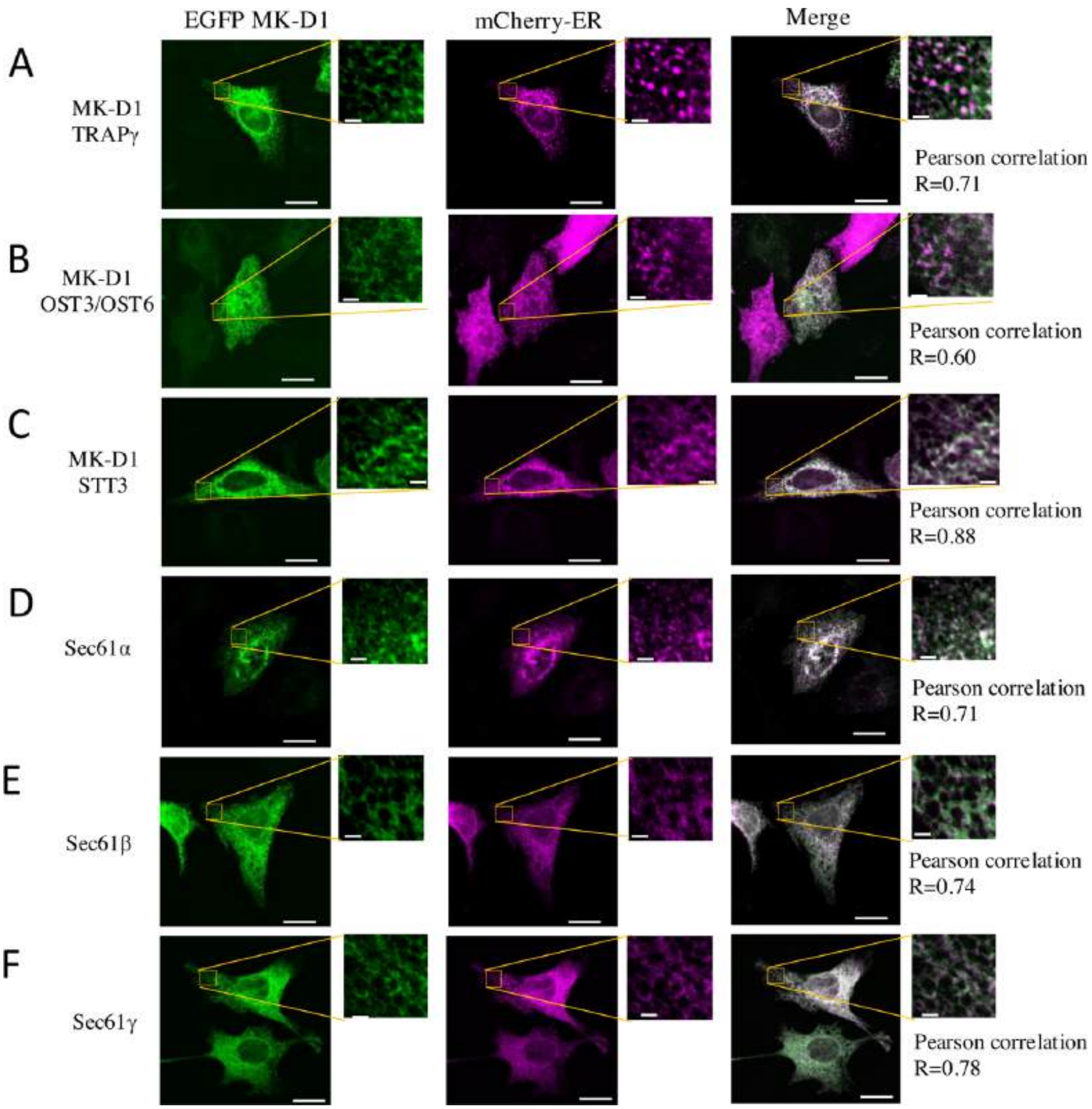

Fig. S7: Pearson correlation evaluation of the colocalization of EGFP-tagged transmembrane proteins with the mCherry-ER marker from Fig. 4 and 5. *A*, TRAPγ; *B*, OST3/OST6; *C*, STT3; *D*, Sec61α; *E*, Sec61β; and *F*, Sec61γ EGFP-tagged MK-D1 proteins. All 6 MK-D1 proteins are directed to the ER, characterized by positive Pearson correlation coefficients (R = 0.60-0.88). Scale bar = 20 μm. Scale bar = 2.3 μm in the magnified images.

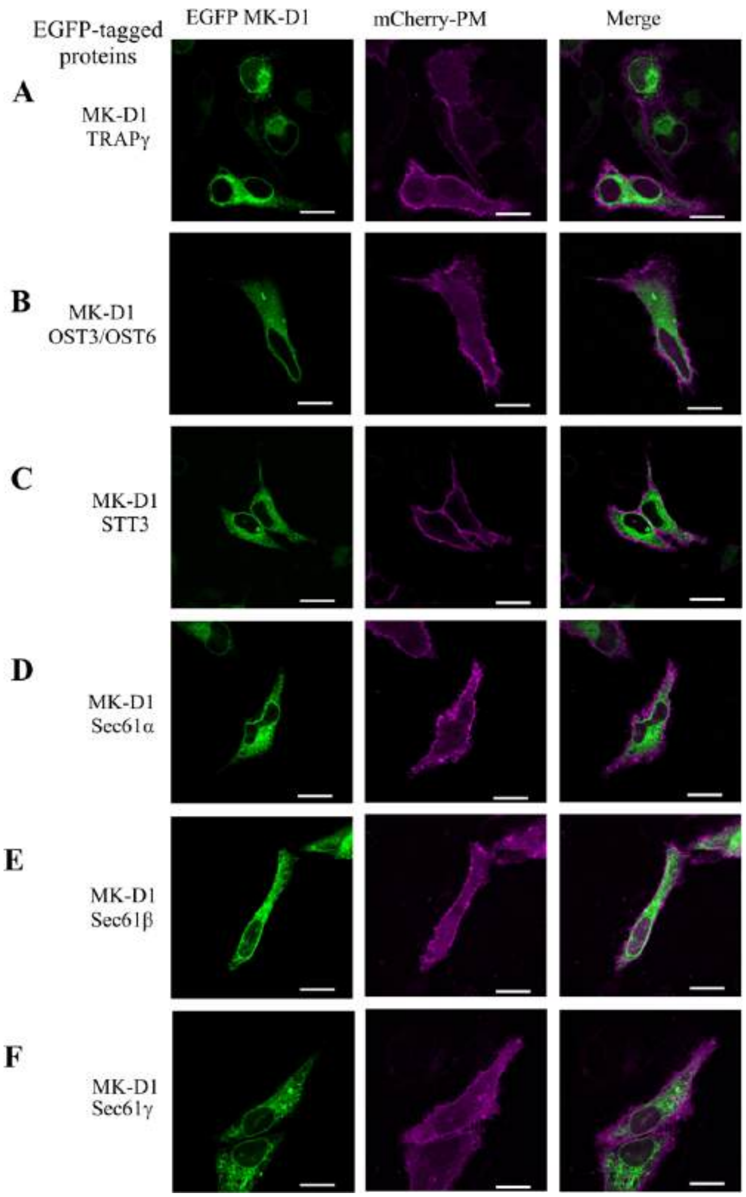

Fig. S8: Colocalization of EGFP-tagged proteins with the mCherry plasma membrane marker as a control for Fig. 4 and 5. *A*, TRAPγ; *B*, OST3/OST6; *C*, STT3; *D*, Sec61α; *E*, Sec61β; and *F*, Sec61γ EGFP-tagged MK-D1 proteins. All 6 MK-D1 EGFP-tagged proteins fluorescence (EGFP, green) fall inside the plasma membrane marker (mCherry-PM, magenta), indicating that these proteins are not associated with the plasma membrane. Scale bar = 20 μm.

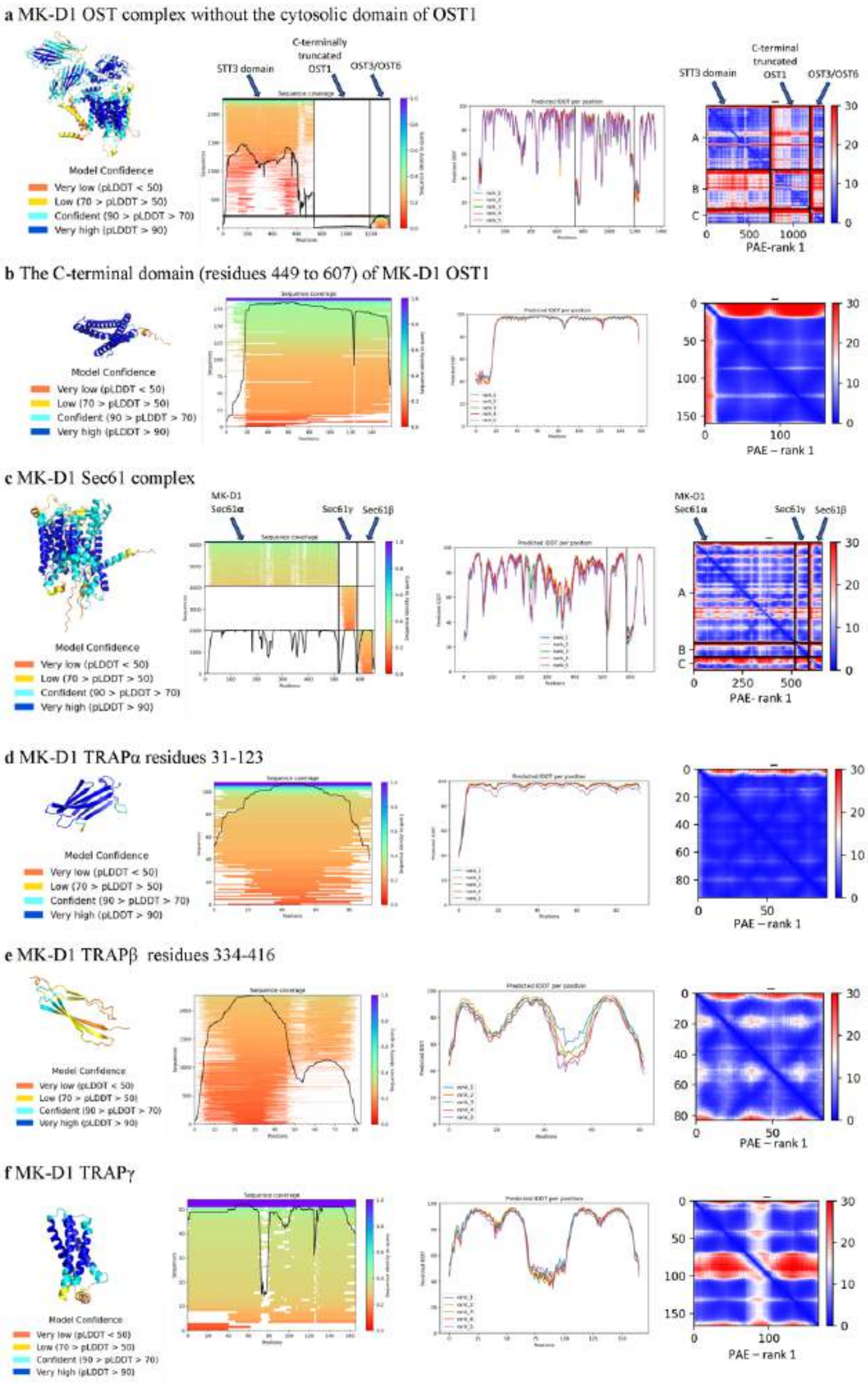

Fig. S9: AF2 statistics for the structure predictions for the segments used to build the MK-D1 translocon complex in Fig. 7*A*.

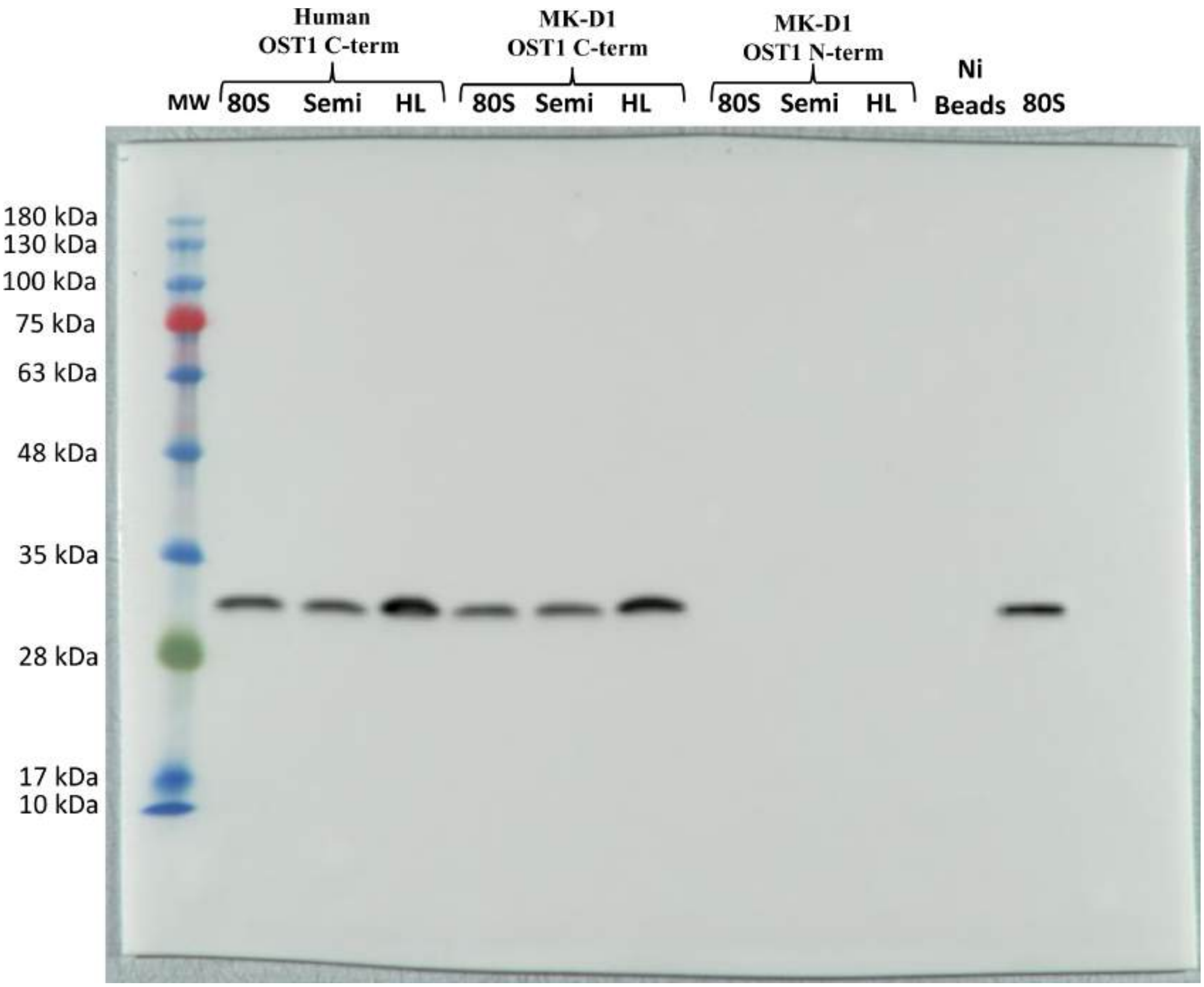

Fig. S10: Full Western blot for the data shown in Fig. 7*D*. Pull-down assay showing interactions of His-tagged versions of the cytosolic domain of MK-D1 OST1(C-term), cytosolic domain of human OST1 (C-term) and the extracellular domain of MK-D1 OST1 (N-term) with various preparations of ribosomes. 80S, highly purified ribosomes; Semi, semi-purified ribosomes; HL, HeLa cell clarified lysate. Beads, control experiment without His-tagged protein, and the final lane the 80S standard. The Western blot is probed with an antibody against ribosomal protein S3 (RPS3). MW, molecular weight markers labelled in kDa.

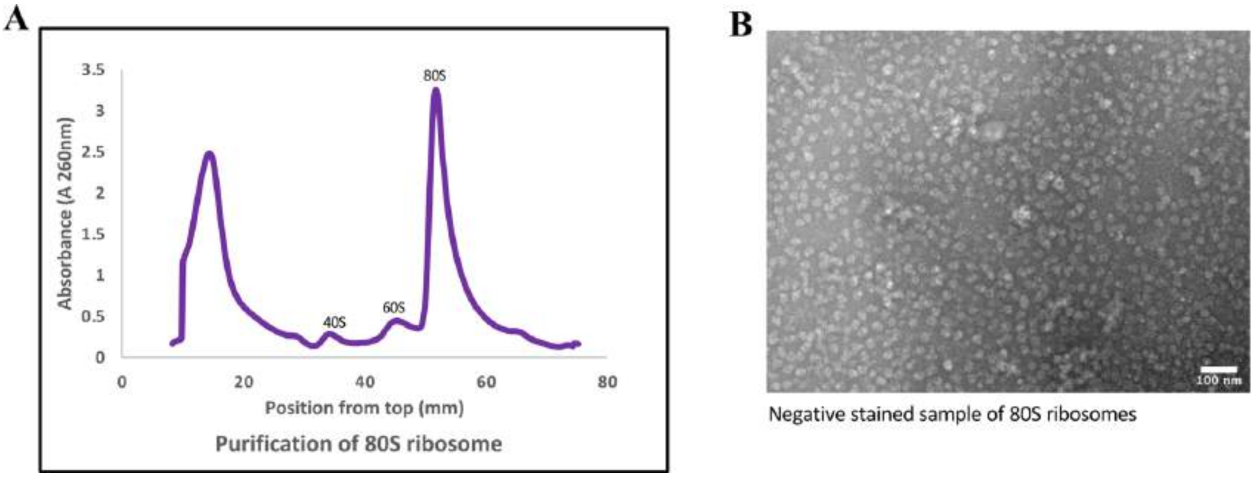

Fig. S11: 80S ribosome preparation. *A*, 10-40% sucrose density gradient harvesting profile. *B*, Electron micrograph of the negatively stained 80S peak.

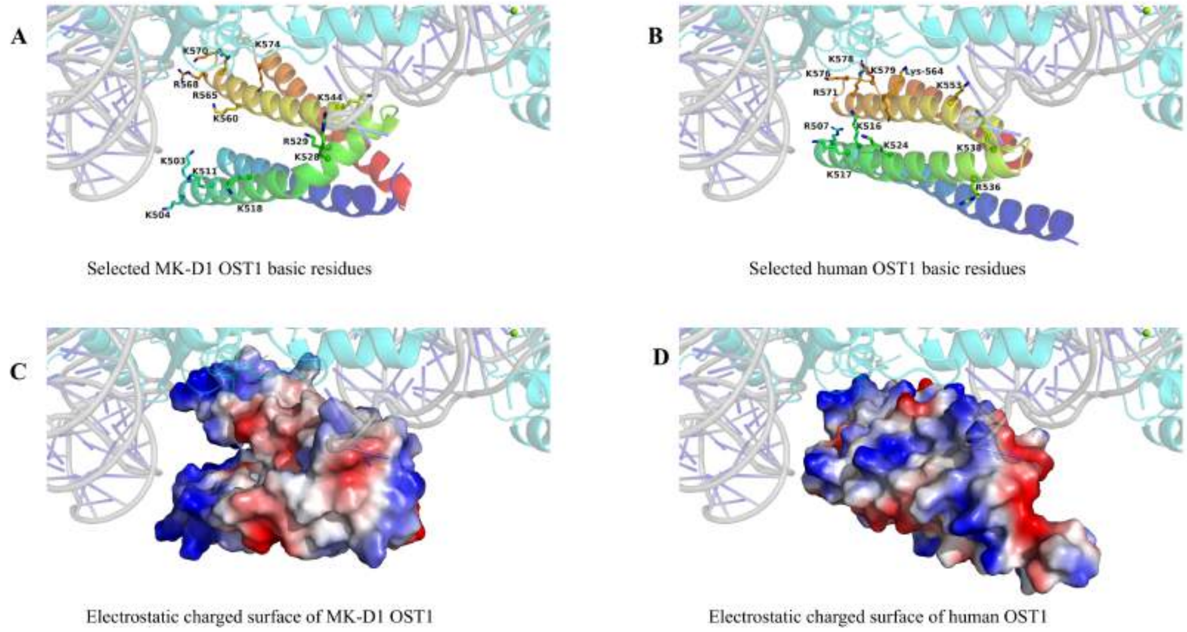

E

MAFFT sequence alignment for the cytosolic domains of MK-D1 OST1 and Human OST1

```
CLUSTAL format alignment by MAFFT (v7.511)

MK-D1   449 RKQRKDYEEGEESFIEQSVPI--------RELTRFVTLIEEKNAILLDIEKADSDLRRKR 500
Human   465 KDPAAEARMKVACITEQVLTLVNKRIGLYRHFDETVNRYKQSR----DISTLNSGKKSLE 520
             :.   :  .    .:  ** :.:        *.:  . *.   ::..    **..  :*. :   .

MK-D1   501 IQKKVYTKTVKNYQNKLKELNEESIPFKRILIETGGQIQSIIQKLDFLEAEKISVKDSVK 560
Human   521 TEHKALTSEIALLQSRLKTEGSDLCDRVSEMQKLDAQVKELVLK-SAVEAERLVAG---K 576
             ::*.  *.  :     *.:**   ..:            :  :  ..*::.::  *  .  :***::  .    *

MK-D1   561 LLKDRYKRGKLPSKAAYERLSSDMIKQLASSQNKIDRYINELRAYII 607
Human   577 LKKDTYIEN--------EKLISGKRQELVT---KIDHILDAL----- 607
            * ** *  ..        *:* *.  ::*.:   ***: :: *
```

Fig. S12: Potential ribosome binding residues on the OST1 cytosolic domain. *A*, Rainbow-colored cartoons of the MK-D1 OST1 cytosolic domain superimposed onto the human OST1 cytosolic domain structure (*B*, PDB 8B6L). Selected basic residues are shown and labeled. *C*,*D* Surface charge representations of *A* and *B*, respectively. A portion of the ribosome is shown with RNA in gray and proteins in cyan. *E*, A structure-based sequence alignment of MK-D1 and human OST1 cytosolic domains. The selected basic residues in *A*,*B* are highlighted in boxes. The residues were selected based on their proximity to the ribosome in the model (*A*) or structure (*B*).

	Human	MK-D1
**OST1**	NP_002941.1	WP_147663064.1
**OST3/6**	NP_001254747.1	QEE15371.1
**STT3**	NP_001265432.1	WP_147662255.1
**TRAP-α**	NP_003135.2	WP_147662165.1
**TRAP-β**	NP_003136.1	WP_147662978.1
**TRAP-γ**	NP_001295126.1	WP_147664586.1
**Sec61α**	NP_001387257.1	WP_162306565.1
**Sec61β**	NP_006799.1	WP_147663065.1
**Sec61γ**	NP_001012474.1	WP_147664346.1
**S-layer**		QEE17131.1
**SRP19**		WP_162306654.1
**SRP54**		QEE15689.1
**SRP docking protein FtsY**		WP_147664347.1

Table S1. NCBI accession codes for the sequences used or discussed in the manuscript
